# Data on medicinal plants used to treat respiratory infections and related symptoms in South Africa

**DOI:** 10.1016/j.dib.2018.10.012

**Published:** 2018-10-09

**Authors:** Sebua Silas Semenya, Alfred Maroyi

**Affiliations:** a Technology Transfer Office, Research Administration and Development Department, University of Limpopo, Private Bag X1106, Sovenga 0727, South Africa; b Medicinal Plants and Economic Development (MPED) Research Centre, Department of Botany, University of Fort Hare, Private Bag X1314, Alice 5700, South Africa

**Keywords:** Chest complaints, Cold, Cough, Fever, Respiratory infections, South Africa, Tuberculosis

## Abstract

We provide details of 306 plant species used to treat and manage respiratory infections and related symptoms in South Africa. About a third of the documented species belong to four plant families, namely the Asteraceae (15.7%), Fabaceae (6.1%), Lamiaceae (5.6%) and Amaryllidaceae (4.6%). An overwhelming majority of documented species are used as medicine to treat tuberculosis (40.2%), cough (36.6%), fever (29.1%), chest complaints (28.8%) and cold (23.2%). The potentially bioactive phytochemical compounds and associated pharmacological properties of the documented plant species are also provided. This data demonstrated strong correlation between phytochemistry, pharmacological properties and medicinal uses of more than three quarters (80.1%) of the documented species used against respiratory infections and related symptoms. Data of this nature can be used to identify research gaps on ethnomedicinal uses, phytochemistry and pharmacological properties of plant species used as herbal medicines.

**Specifications table**

**Table:** 

Subject area	Biology, pharmacology
More specific subject area	Ethnobotany, medicinal plants
Type of data	Table, text file, graph
How data was acquired	Data set was created by selecting articles that meet the pre-determined criteria
Data format	Raw and processed
Experimental factors	Data used in this article were obtained from selected articles that focused on medicinal plants used to treat and manage respiratory infections and related symptoms, and those that highlighting the use of plants for these ailments in South Africa. The pre-determined criteria for the selection: (1) the articles highlighted or focused on medicinal plants used to treat and manage respiratory infections and related symptoms in South Africa, (2) the identity of the utilized species is indicated, (3) the articles were published between 1950 and 2017, (4) the articles are written in English, and (5) articles published as abstract, letters and data that could not be extracted or overlapped with data from other articles were excluded
Experimental features	Data were checked for completeness, reliability and descriptive statistics such as frequencies and percentages were used in the analysis
Data source location	South Africa
Data accessibility	The data are available with this article

**Value of the data**

• The data provide medicinal plants that are a primary source of medicines used against respiratory infections and related symptoms in South Africa.
• The data on medicinal plants used against respiratory infections and related symptoms can be used as a vehicle for ethnopharmacological basis for drug research, pharmaceutical and health products development.
• The data can also be used as a source of information for preserving useful plants implicated as therapy for respiratory infections and related symptoms.
• Data on plants used as medicine for respiratory infections and related symptom in South Africa scattered across literature compiled in one database, and this can be used as baseline data in future research focusing on ethnomedicinal uses, phytochemistry and pharmacological properties of plant species used as herbal medicines

## 1 Data

**Fig. 1:**
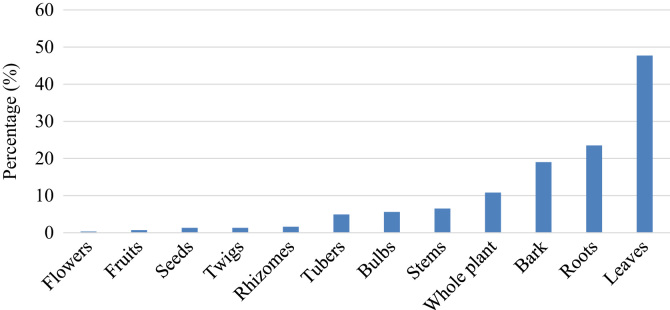
Plant parts used as herbal medicines against respiratory infections and related symptoms in South Africa.

**Fig. 2:**
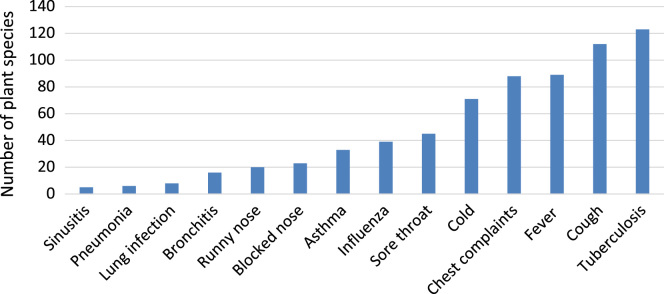
Main respiratory infections and related symptoms treated by at least five plant species.

Plant-based remedies for respiratory infections and related symptoms have been in use in South Africa for several centuries. Respiratory infections particularly asthma, pneumonia, rhinitis, sinusitis and tuberculosis constitute the major causes of mortality and morbidity in both developing and developed countries of the world [1], [2], [3], [4]. Research carried out by York et al. [5] in KwaZulu Natal province, South Africa showed that traditional medicines are widely used in treating various respiratory ailments in this province. Therefore, to explore the importance of traditional medicines in treating and managing respiratory infections and related symptoms throughout South Africa, a database was established documenting plant species, plant parts used, phytochemistry and pharmacological activities of utilized species. A total of 306 plant species belonging to 228 genera and 86 botanical families are used as herbal medicines to treat respiratory infections and related symptoms in South Africa. Appendix A (Supplementary material: Plants used to treat and manage respiratory infections and related symptom in South Africa) provides an alphabetical listing of the plant species. More than half of these species (62.7%) belong to 17 families given in Table 1. Plant families Asteraceae, Fabaceae, Lamiaceae and Amaryllidaceae accounted for the highest number of species (Table 1). About 6.2% of the documented species are exotic to South Africa, 3.6% of the species are of conservation concern and listed in the Red Data List of South African plants, and 1.6% are protected under the South African National Forest Act (Appendix A). Herbs (40.2%), trees (35.6%) and shrubs (23.9%) are the primary sources of the plants used as herbal medicines against respiratory infections and related symptoms in South Africa (Appendix A). Plant organs that are preferred are leaves (47.9%), roots (23.5%), bark (19.0%) and the whole plant (10.8%) (Fig. 1). The main respiratory infections and related symptoms treated by at least five medicinal plant species include tuberculosis, cough, fever, chest complaints, cold, sore throat, influenza, asthma, blocked and runny nose, bronchitis, lung infection, pneumonia and sinusitis, respectively (Fig. 2). Other minor respiratory infections and related symptoms that are also treated by herbal medicines include breathing problems, catarrh, hoarseness, shortness of breath and tonsillitis (Appendix A).

**Table 1:** Botanical families with five or more species used against respiratory infections and related symptoms in South Africa.

**Botanical family**	**Number of species**	**Percentage (%)**
Asteraceae	48	15.7
Fabaceae	19	6.1
Lamiaceae	17	5.6
Amaryllidaceae	14	4.6
Apiaceae	9	2.9
Euphorbiaceae	9	2.9
Myrtaceae	9	2.9
Rutaceae	9	2.9
Geraniaceae	8	2.6
Anacardiaceae	7	2.3
Hyacianthaceae	7	2.3
Solanaceae	7	2.3
Asparagaceae	5	1.6
Combretaceae	5	1.6
Moraceae	5	1.6
Rubiaceae	5	1.6
Xanthorrhoeaceae	5	1.6

## 2 Experimental design, materials and methods

Data presented in the database encompasses plant species that are used to treat and manage respiratory infections and related symptoms in South Africa. Also included in the database are the plant parts used, phytochemistry and pharmacological activities of the documented species. This database was compiled based on selected research articles. In selecting the research articles, several pre-determined criteria were taken into consideration. Firstly, the data was compiled from research articles that focused on medicinal plants used to treat and manage respiratory infections and related symptoms in South Africa. The identity of the utilized herbal medicines was considered to be very important, including details of utilized plant parts. The data was generated from research articles written in English and published between 1950 and 2017. The research articles that were published as abstract, letters and data that could not be extracted or overlapped with data from other articles were excluded.

**Fig. 3:**
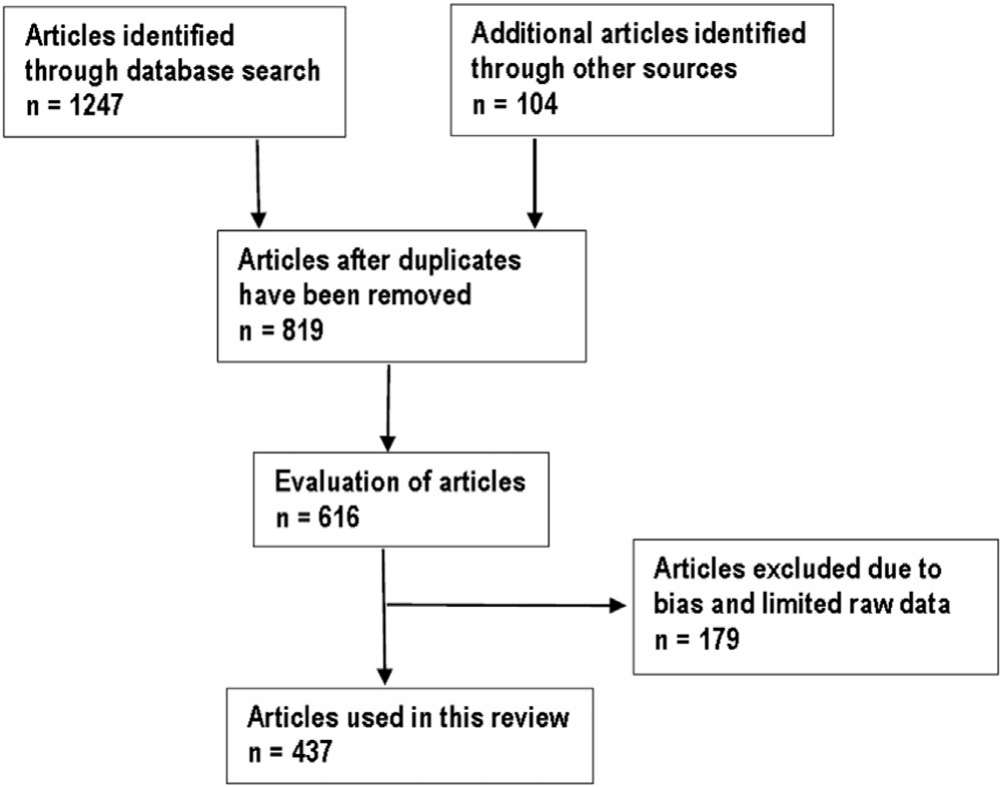
Flow diagram with the number of selected articles.

Literature search for information on plant species that are used to treat and manage respiratory infections and related symptoms in South Africa was carried out from January to December 2017. The information was obtained from the main online scientific sites including Science Direct, SciFinder, Pubmed, Google Scholar, Medline, and SCOPUS. Searches were also undertaken in the library, University of Fort Hare, University of Limpopo and the dissertation search engines like ProQuest, Open-thesis, OATD and EThOS. The species name, botanical families, plant authority, and synonyms were verified using books, journal articles and internet sources such as the International Plant Name Index (www.ipni.org), the Missouri Botanical Garden׳s Tropicos Nomenclatural database (www.tropicos.org) and the Royal Botanic Garden and Missouri Botanic Garden plant name database (www.theplantlist.org). Information on the phytochemistry and pharmacological properties of the documented medicinal plants was generated from the electronic search engines and other literature sources included papers published in international journals, reports from international, regional and national organizations, conference papers, books, theses, websites and other grey literature. A total of 437 articles matched the inclusion criteria and were included in the review (Fig. 3). The data draws heavily on the research articles results published in international journals (329), dissertations and theses (67), books (19), websites (ten), conference reports (nine) and book chapters (three).
