# Supplementary material for: Data on medicinal plants used to treat respiratory infections and related symptoms in South Africa
Source: Data Brief. 2018 Oct 9;21:419–23. doi: 10.1016/j.dib.2018.10.012 (PMC6198089; doi:10.1016/j.dib.2018.10.012)
Supplement: Supplementary file 2 — Supplementary material [file mmc2.docx]

| **Appendix A. Supplementary material** : Plants used to treat and manage respiratory infections and related symptom in South Africa | | | | | | |
| --- | --- | --- | --- | --- | --- | --- |
| **Species name** | **Family** | **Habit** | **Used parts** | **Medicinal uses** | **Bioactive compounds** | **Pharmacological activities** |
| [^ӿ^[*Acacia mearnsii* De Wild.](http://www.ipni.org/ipni/idPlantNameSearch.do;jsessionid=F8BEC04CD6B8EB7FB25238CA21A64BFA?id=470860-1&back_page=%2Fipni%2FeditSimplePlantNameSearch.do%3Bjsessionid%3DF8BEC04CD6B8EB7FB25238CA21A64BFA%3Ffind_wholeName%3DAcacia%2Bmearnsii%2B%26output_format%3Dnormal)](http://www.ipni.org/ipni/idPlantNameSearch.do;jsessionid=F8BEC04CD6B8EB7FB25238CA21A64BFA?id=470860-1&back_page=%2Fipni%2FeditSimplePlantNameSearch.do%3Bjsessionid%3DF8BEC04CD6B8EB7FB25238CA21A64BFA%3Ffind_wholeName%3DAcacia%2Bmearnsii%2B%26output_format%3Dnormal) | Fabaceae | Tree | Bark | Cough, fever, sore throat and tuberculosis (TB) [1] | Epicatechin, epigallocatechin, sistosterol [2] | Ethyl acetate extract of the bark effective against *Staphylococcus aureus*, *Bacillus cereus*, *Shigella flexneri* and *Klebsiella pneumoniae* with MIC values ranging from 1.0 to 10.0 mg/ml [3]. Aqueous, acetone, ethanol and methanol extracts of bark showed antioxidant activities using ABTS and DPPH assays [4] |
| *Acacia karroo* Hayne | Fabaceae | Tree | Leaves, stem | Cold, TB [5,6] | Monoterpenes, sesquiterpenes [7] | Ethyl acetate root extract showed activity against *S.* *aureus* with 35 ± 1.15 mm zone of inhibition [8] |
| *Acalypha peduncularis* E. Mey. ex Meisn. | Euphorbiaceae | Herb | Roots | Chest complaints, cough [9] | - | - |
| [[*Acorus calamus* L.](http://www.ipni.org/ipni/idPlantNameSearch.do?id=84009-1&back_page=%2Fipni%2FeditSimplePlantNameSearch.do%3Ffind_wholeName%3DAcorus%2Bcalamus%26output_format%3Dnormal)](http://www.ipni.org/ipni/idPlantNameSearch.do?id=84009-1&back_page=%2Fipni%2FeditSimplePlantNameSearch.do%3Ffind_wholeName%3DAcorus%2Bcalamus%26output_format%3Dnormal) | Acoraceae | Herb | Roots | Blocked nose, chronic bronchitis, fever [1,10] | Flavonoids, phenolics, proanthocyanidin [11] | Rhizome showed activities with minimum inhibition concentration (MIC) values of 0.25 mg/ml against S*. aureus* and *Bacillus subtilis*. Methanolic extracts of leaves and rhizome showed antioxidant activities using DPPH assay [11] |
| [[*Acokanthera oppositifolia* (Lam.) Codd](http://www.ipni.org/ipni/idPlantNameSearch.do?id=76349-1&back_page=%2Fipni%2FeditSimplePlantNameSearch.do%3Ffind_wholeName%3DAcokanthera%2Boppositifolia%26output_format%3Dnormal)](http://www.ipni.org/ipni/idPlantNameSearch.do?id=76349-1&back_page=%2Fipni%2FeditSimplePlantNameSearch.do%3Ffind_wholeName%3DAcokanthera%2Boppositifolia%26output_format%3Dnormal) | Apocynaceae | Tree | Leaves, roots | Cold [12,13] | Phenolic, flavonoids, flavonols, and proanthocyanidin [14] | Aqueous leaf extracts showed activities against *B.* *subtilis*, *S*. *aureus* and *K. pneumoniae* with MIC values of 6.25 to 12.5 mg/ml (Aremu, 2009). Methanol extract showed antioxidant activities determined by ABTS, DPPH and FRAP methods [14] |
| *Adiantum aethiopicum* L. | Pteridaceae | Herb | Whole plant | Cough [15] | - | - |
| *Agapanthus inapertus* P.Beauv. | Amaryllidaceae | Shrub | Tuber, leaves, stem | Chest complaints, cold, cough, bronchitis, fever, influenza and TB [16,17,18] | Steroidal saponins and sapogenins [19,20] | - |
| [*Agathosma betulina* Berg](http://www.ipni.org/ipni/idPlantNameSearch.do?id=770863-1&back_page=%2Fipni%2FeditSimplePlantNameSearch.do%3Ffind_wholeName%3DAgathosma%2Bbetulina%26output_format%3Dnormal) | Rutaceae | Shrub | Leaves | Cold, fever, influenza [6,21, 22] | Diosphenol, isomenthone, limonene, flavonoids, monoterpene [23] | Essential oils and methanol: dichloromethane (1:1) leaf extracts showed activities against *B*. *cereus*, *K*. *pneumoniae* and *S*. *aureus* with MIC values of 2 to 4 mg/ml [24,25]. Same extracts showed antioxidant activities using ABTS assay [25] |
| *Acanthospermum glabratum* (DC.) Wild | Asteraceae | Herb | Whole plant | Chest pain, cold, cough, fever, runny nose, sore throat [26] | Terpenoids [27] | Dichloromethane:methanol (1:1) of whole plant showed activities against *B*. *cereus* with MIC value of 0.88 mg/mL [28] |
| *Aframomum melegueta* (Rox.) K.Schum | Zingiberaceae | Herb | Roots | TB [18] | Alkaloids, saponins, tannins, terpenes, sterols, glycosides, flavonoids, gingerdione [29,30] | Aqueous leaf extracts showed activities against *K*. *pneumoniae* with zone of inhibition of 6.5 mm [31]. Seed showed antioxidant activity using DPPH assay [32] |
| ^#^*Alepidea amatymbica* Eckl. & Zeyh. | Apiaceae | Herb | Tuber | Fever, cold, cough, TB, influenza, sore throat [13,33,34,35] | Phenolic acid, rosmarinic acid [36,37] | Rhizome dichloromethane, ethanol and petroleum ether extracts exhibited activities against *B*. *subtilis* and *K*. *pneumoniae* with MIC values of 0.39 to 0.78 mg/ml [38] |
| *Alepidea capensis* (P.J.Bergius) R.A.Dyer var. capensis | Apiaceae | Herb | Roots, bark | Chest pain, TB [39] | - | - |
| [^ӿ^[*Allium* *sativum* L.](http://www.ipni.org/ipni/idPlantNameSearch.do?id=528796-1&back_page=%2Fipni%2FeditSimplePlantNameSearch.do%3Ffind_wholeName%3DAllium%2Bsativum%26output_format%3Dnormal)](http://www.ipni.org/ipni/idPlantNameSearch.do?id=528796-1&back_page=%2Fipni%2FeditSimplePlantNameSearch.do%3Ffind_wholeName%3DAllium%2Bsativum%26output_format%3Dnormal) | Amaryllidaceae | Herb | Bulb | Cough, lung infections, TB, fever, sore throat [6,15,40] | Alkaloid, anthraquinones, carbohydrate, flavonoid, glycoside, protein, saponin, tannin and triterpenes [41] | Ethanolic bulb extract showed activities against *Mycobacterium* *tuberculosis*, *S*. *aureus* and *B*. *subtilis* with MIC value of 1 to 3 mg/ml [42,43]. Methanol and water extracts showed antioxidant activities using DPPH method [44] |
| [[*Aloe dichotoma* Masson](http://www.ipni.org/ipni/idPlantNameSearch.do?id=529399-1&back_page=%2Fipni%2FeditSimplePlantNameSearch.do%3Ffind_wholeName%3DAloe%2Bdichotoma%2B%26output_format%3Dnormal)](http://www.ipni.org/ipni/idPlantNameSearch.do?id=529399-1&back_page=%2Fipni%2FeditSimplePlantNameSearch.do%3Ffind_wholeName%3DAloe%2Bdichotoma%2B%26output_format%3Dnormal) | Xanthorrhoeaceae | Tree | Stem | Asthma [21] | - | - |
| *Aloe* *ferox* Mill. | Xanthorrhoeaceae | Shrub | Leaves | Asthma, TB [39,45] | Alkaloids, aloesin, phenols, steroids [46] | Leaf extract showed activities against *B.* *cereus*, *B*. *subtilis*, *S*. *aureus* and *Shigella* *sonnei* [47]. Ethanol and methanol leaf extracts showed antioxidant activity using ABTS and DPPH assays [48] |
| *Aloe marlothii* A. Berger | Xanthorrhoeaceae | Shrub | Leaves | Blocked nose, chest pain, fever [26] | Anthrones, aloin, chromones, aloesin, aloeresin [26] | Leaf dichloromethane and methanol extracts showed activities against *Cryptococcus neoformans* and *K. pneumoniae* with MIC values of 1.0 to 2.7 mg/ml [26] |
| *Anginon difforme* (L.) B.L. Burtt | Apiaceae | Shrub | Not specified | TB [22] | - | - |
| *Andrachne ovalis* (Sond.) Müll. Arg. 18 | Euphorbiaceae | Tree | Roots | Chest complaints [49] | Anthraquinones, flavonoids, glycosides, saponins, steroids[50] | - |
| [[*Anisodontea triloba* (Thunb.) D.M.Bates](http://www.ipni.org/ipni/idPlantNameSearch.do?id=558955-1&back_page=%2Fipni%2FeditSimplePlantNameSearch.do%3Ffind_wholeName%3DAnisodontea%2Btriloba%2B%26output_format%3Dnormal)](http://www.ipni.org/ipni/idPlantNameSearch.do?id=558955-1&back_page=%2Fipni%2FeditSimplePlantNameSearch.do%3Ffind_wholeName%3DAnisodontea%2Btriloba%2B%26output_format%3Dnormal) | Malvaceae | Shrub | Leaves | Asthma, cold [21] | - | - |
| *Annona senegalensis* Pers. | Annonaceae | Tree | Bark, roots, leaves | Fever [51] | Alkaloids, anthocyanins, flavonoid, glycosides, saponins, steroids, tannins, volatile oil [52] | Root bark methanol-methylene chloride (1:1) extracts showed activities against *S*. *aureus* with MIC value of 150 µg/mL [53]. Aqueous methanol and ethyl acetate leaf extracts exhibited antioxidant activities using the DPPH method [53] |
| [*Araucaria bidwillii* Hook.](http://www.ipni.org/ipni/idPlantNameSearch.do?id=133997-3&back_page=%2Fipni%2FeditSimplePlantNameSearch.do%3Ffind_wholeName%3DAraucaria%2Bbidwillii%26output_format%3Dnormal) | Araucariaceae | Tree | Bark | TB [54] | Biflavones [55] | Fruit methanolic extracts showed activities against *B.* *cereus* and *K*. *pneumoniae* with zone of inhibition of 8.7 ± 0.2 mm to 10.2 ± 0.2 mm [56] |
| *Araujia sericifera* Brot | Apocynaceae | Shrub | Rhizome, leaves | TB [6] | Serotonin [57] | - |
| *Arctotis* *arctotoides* (L.f.) O.Hoffm | Asteraceae | erb | Leaves | Catarrh [13] | Sesquiterpene lactones, fernesol derivatives [58,59] | Shoot ethanol extracts showed activity against *B.* *subtilis* [60] |
| ^ӿ^[*Artemisia absinthium* L.](http://www.ipni.org/ipni/idPlantNameSearch.do?id=300106-2&back_page=%2Fipni%2FeditSimplePlantNameSearch.do%3Ffind_wholeName%3DArtemisia%2Babsinthium%26output_format%3Dnormal) | Asteraceae | Herb | Leaves | Cold, cough, influenza [22] | Absinthin, anabsin, arabsin, artabin, artemetin, artenisetin, arthamarin, bisabolen, cadinene, pinene [61] | Essential oil from leaves, stems and flowers showed activities against *S*. *aureus* with zone of inhibition of 20.66 ± 2.61 mm [62] |
| [[*Artemisia afra* Jacq. ex Willd.](http://www.ipni.org/ipni/idPlantNameSearch.do?id=179163-1&back_page=%2Fipni%2FeditSimplePlantNameSearch.do%3Ffind_wholeName%3DArtemisia%2Bafra%2B%26output_format%3Dnormal)](http://www.ipni.org/ipni/idPlantNameSearch.do?id=179163-1&back_page=%2Fipni%2FeditSimplePlantNameSearch.do%3Ffind_wholeName%3DArtemisia%2Bafra%2B%26output_format%3Dnormal) | Asteraceae | Shrub | Leaves | Cold, fever, influenza, asthma, chest pains, cough, TB, blocked nose [6,15,16,18,34] | Acetate, artemisia alcohol, artemisia ketone, ascaridole, borneol, camphor, geraniol, limonene monoterpenoids, terpinen-4-ol [63] | Essential oil from leaves showed activities within 10 minutes at 0.75% concentration against *K. pneumoniae* and within 60 minutes at 1% concentration against for *C*. *neoformans* using time-kill methodology [24] |
| *^ӿ^Artemisia vulgari* L. | Asteraceae | Shrub | Leaves | Fever [22] | Artemisia ketone, caryophyllene, monoterpenes, sabinene, sesquiterpenes, β-pinene [64] | Ethanol extract of stem, root and shoot showed activities against *B*. *subtilis* and *S.* *aureus* with MIC values of 25 to 50 mg/ml [65] |
| *Asclepias crispa* P.J. Bergius | Apocynaceae | Herb | Roots | Chest complaints [22] | - | - |
| [*Asclepias* *fruticosa* L.](http://www.ipni.org/ipni/idPlantNameSearch.do?id=94302-1&back_page=%2Fipni%2FeditSimplePlantNameSearch.do%3Ffind_wholeName%3DAsclepias%2Bfruticosa%26output_format%3Dnormal) | Apocynaceae | Shrub | Roots | TB, cough [66,67] | Cardenolide glycosides, steroidal, kaempferol, isorhamenetin, rutin [68,69] | Hexane and methanol fruits extracts showed activities against *Pseudomonas aeruginosa* with MIC  value of 31 µg/ml [70] |
| *Aspalathus cordata* (L.) R.Dahlgren | Fabaceae | Shrub | Leaves | Asthma [15] | - | - |
| [*Asparagus falcatu*s L.](http://www.ipni.org/ipni/idPlantNameSearch.do?id=531097-1&back_page=%2Fipni%2FeditSimplePlantNameSearch.do%3Ffind_wholeName%3DAsparagus%2Bfalcatus%26output_format%3Dnormal) | Asparagaceae | Shrub | Roots | TB [71] | Capsoneoxanthin [72] | Leaf ethanol extract showed activities against *K*. *pneumoniae*, *Mycobacterium aurum* and *S*. *aureus* with MIC values of 0.39 to 6.25 mg/mL [73] |
| *Asparagus africanus* Lam 31 | Asparagaceae | Shrub | Leaves | TB [6] | Antiprotozoal, steroidal saponins [74,75] | Leaf ethanol extract showed activities against K. pneumoniae, S. aureus, *M. aurum* and *M. tuberculosis* with MIC of 0.39 to 6.25 mg/mL [76] |
| *Asparagus laricinus* Burch. | Asparagaceae | Shrub | Roots | TB, chest complaints [15,22] | Flavonoids, glycosides, phlobatannins, saponins, steroids, tannins, terpenoids [77] | Leaf extracts showed activities against *S*. *aureus* and *B*. *subtilis* with MIC values of 01 mg/ml [77]. Same authors reported antioxidant activities using DPPH assay. |
| *Aster harveyanus* Kuntze subsp. nyikensis Lippert | Asteraceae | Herb | Leaves, roots | Cough, TB [35] | - | - |
| *Aster boekerianus* L. | Asteraceae | Tree | Not specified | Chest pain, TB [34] | - | Aqueous leaf extracts showed activities against *S. aureus* and *K*. *pneumoniae* with MIC value of 0.5 mg/ml [78] |
| [[*Ballota africana* Benth.](http://www.ipni.org/ipni/idPlantNameSearch.do?id=444921-1&back_page=%2Fipni%2FeditSimplePlantNameSearch.do%3Ffind_wholeName%3DBallota%2Bafricana%2B%26output_format%3Dnormal)](http://www.ipni.org/ipni/idPlantNameSearch.do?id=444921-1&back_page=%2Fipni%2FeditSimplePlantNameSearch.do%3Ffind_wholeName%3DBallota%2Bafricana%2B%26output_format%3Dnormal) | Lamiaceae | Shrub | Roots, leaves | Cold, fever, influenza, cough [15,21,22,40] | Hispanolone [79] | Leaf dichloromethane extract showed activities against *Streptococcus pyogenes, K. pneumoniae* and *S. aureus* with MIC values of 0.39 to 6.25 mg/mL [80] |
| [*Bauhinia galpinii* N.E.Br.](http://www.ipni.org/ipni/idPlantNameSearch.do?id=481322-1&back_page=%2Fipni%2FeditSimplePlantNameSearch.do%3Ffind_wholeName%3DBauhinia%2Bgalpinii%2B%26output_format%3Dnormal) | Fabaceae | Shrub | Tuber | Pneumonia [81] | Flavonoid glycosides [82] | Hexane, dichloromethane, ethyl acetate and butanol leaf extracts showed activities against *S. aureus* with MIC values of 39 to 156 µg/ml [83]. Antioxidant activity have been reported using assay [82] |
| *Berchemia discolor* (Klotzsch) Hemsl. | Rhamnaceae | Tree | Bark, roots | TB [66] | Prenylated flavonoids [84] | Acetone bark extract showed activities against *M. tuberculosis* with MIC value of 12.5 mg/ml [66] |
| *Bersama lucens* (Hochst.) Szyszyl. | Melianthaceae | Shrub | Bark, roots | Headache [13] | Triterpenoids [85] | Bark ethanol extract showed activities against *B. subtilis*, *K. pneumoniae* and *S. aureus* with MIC value of 3.13 m g/mL. [86] |
| *Bidens pilosa* L. | Asteraceae | Shrub | Leaves, bark | TB [6] | Flavonoids, phenolic, terpenoids, phenylpropanoids, aromatics, porphyrins [87,88] | Leaf extract showed activities against *M. tuberculosis* [89] |
| *Boophone haemanthifolia* F. M.Leight. | Amaryllidaceae | Herb | Bulb | Asthma [21] | - | - |
| *Boophone disticha* (L.f.) Herb. | Amaryllidaceae | Herb | Leaves, roots | Cough [35] | Alkaloids, crinanes, buphanidrine, distichamine [90] | Bulb ethanolic extract showed activities against *K. pneumoniae* and *S. aureus* with MIC value of 0.06 mg/ml [90] |
| *Boscia oleoides* (Burch. ex DC.) Toelken | Capparaceae | Tree | Leaves | Cold [15] | - | - |
| ^#^*Bowiea volubilis* Harv.ex.Hook.f. subsp. volubilis | Hyacinthaceae | Herb | Bulb, roots | Asthma [13,16] | Bufadienolides, glucobovoside [91] | Bulb ethanolic extract showed activities against *B. subtilis, K. pneumoniae* and *S. aureus* with MIC value of 3.13 mg/ml [86] |
| *Bridelia cathartica* Bertol.f. subsp. cathartica | Euphorbiaceae | Tree | Leaves | Cold, cough, runny nose [26] | Anthocyanins, flavonoids, tannins [92] | Hexane extract of bark and root showed activities against *Mycobacterium smegmatis* and *S. aureus* with MIC values of 7.5 to 15.0 mg/ml [70] |
| [*Brachylaena discolor* DC.](http://www.ipni.org/ipni/idPlantNameSearch.do?id=186090-1&back_page=%2Fipni%2FeditSimplePlantNameSearch.do%3Ffind_wholeName%3DBrachylaena%2Bdiscolor%2B%26output_format%3Dnormal) | Asteraceae | Tree | Leaves | Cough, fever, blocked and runny nose [26,93] | Alkaloids, flavonoids, phlobatannins, saponins, tannins, terpenoids [94] | Methanol and aqueous extracts of leaves exhibited antioxidant activities using DPPH assay [94]. Leaf acetone extract showed activities against *S. aureus* with MIC value of 1.25 mg/ml |
| *Brachylaena elliptica* (Thunb.) DC. | Asteraceae | Tree | Leaves | Sore throat, TB [15,95] | Alkaloids, flavanoids, flavanols, phenols, proanthocyanidins, saponins, tannins [96] | Aqueous and ethanol leaf extracts showed activities against *P. aeruginosa* and *S. pyrogenes* with MIC values of 2.5 mg/ml [96] . Same author found that aqueous and ethanol leaf extracts exhibited antioxidant activity using DPPH assay |
| *Brachylaena ilicifolia* (Lam.) Phill. & Schweick. | Asteraceae | Tree | Leaves | Asthma, cough, sore throat [106] | Alkaloids, flavanoids, flavanols, phenols, proanthocyanidins, saponins, tannins [96] | Aqueous and ethanol leaf extracts showed activities against *P. aeruginosa* and *S. pyrogenes* with MIC values of 2.5 2.5 mg/ml [96]. Same author found that aqueous and ethanol leaf extracts exhibited antioxidant activity using DPPH assay |
| *Bridelia micrantha* Hochst. Baill | Euphorbiaceae | Tree | Bark | TB [66] | Carotenoids, flavonoids, flavones, tannins, terpenoids [97] | Acetone bark extract showed activities against *M. tuberculosis* with MIC value of 25 g/mL [66] |
| [[*Buddleja saligna* Willd.](http://www.ipni.org/ipni/idPlantNameSearch.do?id=545876-1&back_page=%2Fipni%2FeditSimplePlantNameSearch.do%3Ffind_wholeName%3DBuddleja%2Bsaligna%2B%26output_format%3Dnormal)](http://www.ipni.org/ipni/idPlantNameSearch.do?id=545876-1&back_page=%2Fipni%2FeditSimplePlantNameSearch.do%3Ffind_wholeName%3DBuddleja%2Bsaligna%2B%26output_format%3Dnormal) | Buddlejaceae | Tree | Leaves | Cold, cough [81] | Terpenoids [98] | Methanol stem extract showed activities against *B. cereus* and *S. pyrogens* with MIC value of 2.25 mg/ml [99]. Methanol leaf and stem extracts showed antioxidant activities using ABTS and DPPH assays [99] |
| *Buddleja salviifolia* (L.) Lam. | Buddlejaceae | Tree | Leaves, stem | Fever [16] | Acteoside, quercetin [100] | Ethyl acetate, aqueous and methanol extracts exhibited activities against *B. subtilis* and *S. aureus* with MIC values ranging from 390.0 to 780.0 µg/mL [100] |
| *Bulbine narcissifolia Salm*-Dyck | Xanthorrhoeaceae | Herb | Not specified | Cough, TB [34] | Acetosyringonen, chrysophanol, knipholone, isoknipholone [101] | Acetate root extract exhibited activity against *B. subtilis* and *S. aureus* with MIC value of 1.0 mg/ml [102] |
| *^ӿ^*[*Cannabis sativa L.*](http://www.ipni.org/ipni/idPlantNameSearch.do?id=149763-3&back_page=%2Fipni%2FeditSimplePlantNameSearch.do%3Ffind_wholeName%3DCannabis%2Bsativa%26output_format%3Dnormal) | Cannabaceae | Herb | Leaves | Asthma, cough, TB [6,9,16,18] | Flavonoids, phenols, quinones, saponins, tannins [103] | Ethanol leaf extract showed activity against *S. aureus* and *P. aeruginosa* with zone of inhibition of 10.3 to 24.1 mm [104] |
| *Capparis brassii* DC. | Capparaceae | Shrub | Roots | Cough [8] | - | Methanol root bark extract showed activity against *M. tuberculosis* with MIC value of 1250 μg/ml [105] |
| [[*Capparis* *sepiaria* Wall.](http://www.ipni.org/ipni/idPlantNameSearch.do?id=146770-1&back_page=%2Fipni%2FeditSimplePlantNameSearch.do%3Ffind_wholeName%3DCapparis%2Bsepiaria%2B%26output_format%3Dnormal)](http://www.ipni.org/ipni/idPlantNameSearch.do?id=146770-1&back_page=%2Fipni%2FeditSimplePlantNameSearch.do%3Ffind_wholeName%3DCapparis%2Bsepiaria%2B%26output_format%3Dnormal) | Capparaceae | Shrub | Bark | Cough [106] | Alkaloids, flavonoids, saponins, tannins [107] | Aqueous, ethanolic and hexane leaf extracts showed antioxidant activities using ferric thiocynate method [107] |
| [[*Capparis tomentosa* Lam.](http://www.ipni.org/ipni/idPlantNameSearch.do?id=146824-1&back_page=%2Fipni%2FeditSimplePlantNameSearch.do%3Ffind_wholeName%3DCapparis%2Btomentosa%26output_format%3Dnormal)](http://www.ipni.org/ipni/idPlantNameSearch.do?id=146824-1&back_page=%2Fipni%2FeditSimplePlantNameSearch.do%3Ffind_wholeName%3DCapparis%2Btomentosa%26output_format%3Dnormal) | Capparaceae | Tree | Bark, stem, roots | Chest complaints, sore throat [35, 108, 109] | Nerolidol, ocimene, terpineol, terpineol [110] | Root ethanol extract showed activities against *B. subtilis* and *K*. *pneumoniae* with MIC values of 3.13 mg/ml [86] |
| **Capsicum* *fructescens* L. | Solanaceae | Shrub | Fruits | Fever, TB [33,111] | Alkaloids, anthraquinones, flavonoids, phenols, terpenes [112] | Methanol and aqueous fruit extracts showed activities against *S. aureus* with MIC value of 0.25 mg/ml [113] |
| **Carica* *papaya* L. | Caricaceae | Tree | Leaves | TB [18, 66] | Alkaloids, cynogenetic, flavonoids, phenolic [114] | Leaf acetone extract showed activities against *S. pyogenase* and *P. aeruginosa* with zone of inhibition of 8 to 10 mm and MIC values of 250 mg/ml against *S. aureus* and *S. pneumonia* [115] |
| *Carisa edulis* Forssk. Vahl | Apocynaceae | Tree | Leaves | TB [66] | Cardioglycosides, carissin, quebrachytol [116] | Methanol roots extracts exhibited activity against *M. tuberculosis* and *Mycobacteria kansasii* with MIC value of 0.5mg/ml [117] |
| *Carpobrotus edulis* (L) Bolus | Aizoaceae | Herb | Leaves | TB, sore throat, lung infections [6, 15,22,118,119] | Alkaloids, flavonoids, flavonols, phenolics, proanthocyanidins, saponins, tannins [120] | Aqueous leaf extract showed activity against *S. aureus* and *P. aeruginosa* with MIC values of 4.0 to 6.5 mg/ml [121]. Same authors found that aqueous leaf extract possess antioxidant activity using DPPH method |
| [*Cassine* *transvaalensis* (Burtt Davy) Codd](http://www.ipni.org/ipni/idPlantNameSearch.do?id=160123-1&back_page=%2Fipni%2FeditSimplePlantNameSearch.do%3Ffind_wholeName%3DCassine%2Btransvaalensis%26output_format%3Dnormal) | Celastraceae | Tree | Bark | Cough [66] | Flavonoids, phenolic, tannin [122] | Bark extract showed activities against *S. aureus* and *Staphylococcus epidermidis* with MIC value of 0.02 μg/ml [123]. Water and ethanol extracts of roots showed antioxidant activity using DPPH method [124] |
| *Cassia petersiana* Bolle | Fabaceae | Tree | Bark | TB [66] | Alkaloids, anthraquinones, flavonoids, steroids, tannins, triterpenes [125] | - |
| *Cassytha ciliolate* Nees | Lauraceae | Shrub | Whole plant | Fever [22] | - | - |
| ^α^*Catha edulis* (Vahl.) Endl. | Celastraceae | Tree | Leaves | Asthma and cough [15] | Alkaloids, amino acids, cathinone, glycosides, tannins [126] | Ethanol and water leaf extracts showed activities against *S. pyogenes* with MIC values of 0.39 mg/ml [80] |
| *^ӿ^Centaurea* *benedicta* (L.) L. | Asteraceae | Herb | Whole plant | Cough, hoarseness [15] | - | - |
| *Centella* *coriacea* L. | Apiaceae | Herb | Leaves | TB [6] | - | - |
| *Chironia* *baccifera* L. | Gentianaceae | Shrub | Root | Sore throat, TB [1,18,127] | Tannins, saponins [127] | Ethanol extracts of leaves and stems showed activities against *S. aureus*, *P. aeruginosa* and *M. smegmatis* with MIC value of 1. 25 mg/ml [127] |
| [*Chrysocoma* *ciliata* L.](http://www.ipni.org/ipni/idPlantNameSearch.do?id=193897-1&back_page=%2Fipni%2FeditSimplePlantNameSearch.do%3Ffind_wholeName%3DChrysocoma%2Bciliata%2B%26output_format%3Dnormal) | Asteraceae | Shrub | Leaves | Cold [21] | Monoterpenes, sesquiterpenes [128] | Leaf acetone, ethanol and methanol showed activities against *S. aereus*, *S. epidermidus*, *B. cereus* and *Streptococcus faecalis* with MIC value of 0.1 mg/ml [128] |
| [*Cissampelos* *mucronata* A. Rich.](http://www.ipni.org/ipni/idPlantNameSearch.do?id=580406-1&back_page=%2Fipni%2FeditSimplePlantNameSearch.do%3Ffind_wholeName%3DCissampelos%2Bmucronata%26output_format%3Dnormal) | Menispermaceae | Herb | Roots | Chest problems, influenza [129] | Alkaloids [130] | - |
| *Cissampelos capensis* L.f. | Menispermaceae | Tree | Roots | Sinusitis, TB [16] | Alkaloids, flavonoids [131] | Methanol aerial shoot and root extracts showed activities against *B. cereus* with 45 mm zone of inhibition [131] |
| [*Cissampelos torulosa* E.Mey. ex Harv. & Sond.](http://www.ipni.org/ipni/idPlantNameSearch.do?id=580464-1&back_page=%2Fipni%2FeditSimplePlantNameSearch.do%3Ffind_wholeName%3DCissampelos%2Btorulosa%26output_format%3Dnormal) | Menispermaceae | Herb | Leaves, stem | Sore throat [132] | - | Acetone, hexane and methanol leaf extracts showed activities against B. cereus and S. aureus with MIC values of 412 mg/ml [133] |
| *^ӿ^Cirsium* *vulgare* (Savi) Ten. | Asteraceae | Herb | Whole plant | Cough, hoarseness [15] | Flavonoids, sterols, phenolic acids, tannins, triterpens [134] | Methanol extract of inflorescences showed antioxidant activity using ABTS assay [135] |
| **Citrus* *lemon* (L.) Burm.f. | Rutaceae | Tree | Leaves | Cough, blocked nose, TB [18,26,66,136] | Flavonoids, glycosides, phlobatannins, saponins, tannins [137] | Aqueous fruit extract showed activity against *S. aureus*, *S. epidermidis*, *S. pneumonia* and *S. pyogenes* with inhibition zone ranging from 13 to 30 mm [138] |
| *Clausena* *anisata* (Willd.) Hook. F. ex Benth. | Rutaceae | Tree | Leaves | Fever, cough, blocked and runny nose, sore throat [1,26,106] | Alkaloids, flavonoids, glycosides, saponins, steroids, tannins [139] | Acetone leaf extract showed activity against *S. pyogenes*, *S. aureus* and *B*. *cereus* with MIC values of 0.1 to 0.5 mg/ml [111] |
| *Clausena africana* Hook | Rutaceae | Tree | Leaves, bark | Chest complaints, TB [6] | - | - |
| *Clematis brachiata* Thunb. | Ranunculaceae | Herb | Stem, leaves | Blocked and runny nose, chest pain, cough [26,33,140] | Flavonoids, glycosidic, phenols, saponin, tannin, terpenoids [141] | Acetone and methanol leaves extracts showed activities against *B. cereus*, *K. pneumoniae*, *S.* *epidermidis* and *S. faecalis* with MIC values ranging from 1.0 to 3.0 mg/ml [141] |
| [*Clerodendrum* *glabrum* E.Mey.](http://www.ipni.org/ipni/idPlantNameSearch.do?id=862099-1&back_page=%2Fipni%2FeditSimplePlantNameSearch.do%3Ffind_wholeName%3DClerodendrum%2Bglabrum%2B%2B%26output_format%3Dnormal) | Lamiaceae | Tree | Leaves | Chest complaints, cold, fever, sore throat [132] | Triterpenes [142] | Hexane leaf extract showed activities against *S. aureus* with MIC values of 0.8 mg/ml [142] |
| ^#^[*Clivia* *miniata* Regel](http://www.ipni.org/ipni/idPlantNameSearch.do?id=63633-1&back_page=%2Fipni%2FeditSimplePlantNameSearch.do%3Ffind_wholeName%3DClivia%2Bminiata%26output_format%3Dnormal) [var. miniata](http://redlist.sanbi.org/species.php?species=2081-5) | Amaryllidaceae | Herb | Tuber | Fever [143] | Alkaloids [144] | - |
| [*Colophospermum* *mopane* (J.Kirk ex Benth.) J.Léonard](http://www.ipni.org/ipni/idPlantNameSearch.do?id=486795-1&back_page=%2Fipni%2FeditSimplePlantNameSearch.do%3Ffind_wholeName%3DColophospermum%2Bmopane%26output_format%3Dnormal) | Fabaceae | Tree | Bark, leaves | Cough [145] | Flavonoids [146] | Leaf extract showed activities against *S. aureus* with MIC values of 125 μg/ml [147] |
| *Combretum apiculatum* Sond. | Combretaceae | Tree | Bark, leaves, roots | Chest pain [13] | Alpinetin, pinocembrin, flavokawain [148] | Leaf methanol extract showed antioxidant activity using DPPH method [82] |
| *Combretum hereroense* Schinz. | Combretaceae | Tree | Bark, seeds | TB [18] | Cardiac glycosides, flavonoids, steroids, tannins, terpenes, terpenoids [149] | Acetone leaf extract showed activities against *M. smegmatis* with MIC value of 0.47 mg/ml [149] |
| *Combretum erythrophyllum* (Burch) Sond. | Combretaceae | Tree | Leaves | TB [66] | Triterpenoids [150] | Carbon tetrachloride and chloroform leaf extracts showed activities against *S. aureus* with MIC values of 0.78 mg/ml [151] |
| *Combretum molle* R. Br. ex G. Don | Combretaceae | Tree | Leaves | Cold, fever, blocked nose, cough, TB [1,26,152] | Alkaloids, arjunolic acid, mollic acid glucosides, saponin, saponins, stilbenes, tannins, triterpene, triterpenoid glycosides, trypetitepene [153] | Acetone bark extract showed activity against *S. pyogenes* with MIC value of 0.156 mg/ml [153] |
| [*Conyza scabrida* DC.](http://www.ipni.org/ipni/idPlantNameSearch.do?id=197697-1&back_page=%2Fipni%2FeditSimplePlantNameSearch.do%3Ffind_wholeName%3DConyza%2Bscabrida%2B%26output_format%3Dnormal) | Asteraceae | Shrub | Leaves | Chest complaint, cold, fever [15,21,40] | Cardiac glycosides, saponins, tannin [154] | Methanol leaf extract showed activity against *M. smegmatis* with MIC value of 0.315 mg/ml [127] |
| *Corymbia citriodora* L. | Myrtaceae | Tree | Leaves | TB [6] | Monoterpene, sesquiterpene [155] | Essential oil from leaves showed activities with zone of inhibition of 12.1 ± 1.9 to 32.7 ± 5.1 mm against *S. aureus* [156] |
| *Cotyledon orbiculata* L. | Crassulaceae | Shrub | Leaves | Sore throat [15] | Bufadienolides, saponins, tannins, triterpene steroids [157] | Dichloromethane, ethanol and petroleum ether extracts of leaves showed activities against *B. subtilis*, *S. aureus* and *K. pneumonia* with MIC values of 3.13 mg/ml. Leaf extracts exhibited antioxidant activity using thiobarbituric acid assay [157] |
| *Crassula ericoides* Harv. | Crassulaceae | Shrub | Whole plant | Fever [15] | - | - |
| *Crassula muscosa* L. | Crassulaceae | Tree | Whole plant | Fever [15] | - | - |
| [*Crinum bulbispermum* (Burm.f.) Milne-Redh. & Schweick.](http://www.ipni.org/ipni/idPlantNameSearch.do?id=63828-1&back_page=%2Fipni%2FeditSimplePlantNameSearch.do%3Ffind_wholeName%3DCrinum%2Bbulbispermum%26output_format%3Dnormal) | Amaryllidaceae | Herb | Roots | Cough [17] | Alkaloid [158] |  |
| [*Crinum macowanii* Baker](http://www.ipni.org/ipni/idPlantNameSearch.do?id=63973-1&back_page=%2Fipni%2FeditSimplePlantNameSearch.do%3Ffind_wholeName%3DCrinum%2Bmacowanii%2B%26output_format%3Dnormal) | Amaryllidaceae | Herb | Bulb | Cough, fever [67,81] | Alkaloid [158] |  |
| [*Croton gratissimus* Burch.](http://www.ipni.org/ipni/idPlantNameSearch.do?id=342614-1&back_page=%2Fipni%2FeditSimplePlantNameSearch.do%3Ffind_wholeName%3DCroton%2Bgratissimus%26output_format%3Dnormal) | Euphorbiaceae | Tree | Leaves, roots | Fever, cold, cough [1,49] | Cembranolides [159] | Essential oil from leaves exhibited activity against *B. cereus* and *S. aureus* with MIC values of 0.6 to 1.3 mg/ml [160] |
| [*Croton sylvaticus* Hochst.](http://www.ipni.org/ipni/idPlantNameSearch.do?id=343591-1&back_page=%2Fipni%2FeditSimplePlantNameSearch.do%3Ffind_wholeName%3DCroton%2Bsylvaticus%26output_format%3Dnormal) | Euphorbiaceae | Tree | Bark | TB [161] | Alkaloids, anthraquinones, diterpenoids, flavonoids, phenolics, sterols, tannins, terpenoids [162] | Methanol stem bark extract showed activities against *B. subtillis* with MIC value of 10 mg/mL [162] |
| *Cryptocarya latifolia* Sond. | Lauraceae | Tree | Bark | Chest complaints, TB [49] | Diterpenoids, essential oils, monoterpenoids, sesquiterpenoids [163] | Acetone and water bark extracts showed activities against *M. tuberculosis* (Lall and Meyer, 1999). Fruit and leaf extract showed antioxidant activity using DPPH assay [163] |
| [*Cucumis hirsutus* Sond.](http://www.ipni.org/ipni/idPlantNameSearch.do?id=292208-1&back_page=%2Fipni%2FeditSimplePlantNameSearch.do%3Ffind_wholeName%3DCucumis%2Bhirsutus%26output_format%3Dnormal) | Cucurbitaceae | Herb | Roots | Cough [1,9] | - | Petroleum ether leaf extract showed activity against *E. coli*, *K. pneumoniae*, *B. subtilis* and *S. aureus* with MIC vales of 0.098 to 0.26 mg/ml [164] |
| [*Cussonia spicata* Thunb.](http://www.ipni.org/ipni/idPlantNameSearch.do?id=90185-1&back_page=%2Fipni%2FeditSimplePlantNameSearch.do%3Ffind_wholeName%3DCussonia%2Bspicata%2B%26output_format%3Dnormal) | Araliaceae | Tree | Bark, leaves | Cough [81] | - | Ethanolic and ethyl acetate bark and root extracts showed activities against *S. aureus* with MIC value of 12.5 mg/ml [165] |
| [*Cymbopogon marginatus* Stapf ex Burtt Davy](http://www.ipni.org/ipni/idPlantNameSearch.do?id=396960-1&back_page=%2Fipni%2FeditSimplePlantNameSearch.do%3Ffind_wholeName%3DCymbopogon%2Bmarginatus%2B%26output_format%3Dnormal). | Poaceae | Herb | Leaves | Fever [108] | - | - |
| *Cynodon dactylon* L. | Poaceae | Herb | Rhizome | Cough [15] | Alkaloids, flavonoids, glycosides, tannins, terpenoids, phenolics [166] | Acetone and methanol whole plant extracts showed activity against *B. cereus* and *B. subtilis* with zone of inhibition of 7.0 to 8.0 mm [166] |
| *Cyperus articulatus* L. | Cyperaceae | Herb | Bulb | Blocked nose, chest pain, cold, cough, shortness of breath [26] | Corymbolone [167] | Essential oil extract showed activity against *B. cereus*, *Bacillus megaterium*, *S. pyogene* and *S. epidermidis* with MIC value of 0.1 to 0.2 μg/ml [167] |
| *^ӿ^Datura stramonium* L. | Solanaceae | Shrub | Leaves | Chest ailments, lung infections [168] | Alkaloids, glycosides, saponins, tannins [170] | Petroleum ether stem extract showed activity against *S. pyogenes* and *S. aureus* with MIC values of 0.195 to 0.78 mg/ml [80] |
| **Daucus* *carota* L. | Apiaceae | Herb | Leaves, fruits | TB [5] | Alkaloids, carbohydrates, coumarin, essential oil, flavonoids, phenols, terpenoid [171] | Ethanol seed extract showed activity against *B. subtilis* and *S. aureus* with minimum bactericidal concentration (MBC) value of 3.125 mg/ml (Pavlyuk et al., 2015). Methanol seed extract showed antioxidant activities using DPPH methods [172] |
| [*Dichrostachys cinerea* (L.) Wight & Arn.](http://www.ipni.org/ipni/idPlantNameSearch.do?id=492423-1&back_page=%2Fipni%2FeditSimplePlantNameSearch.do%3Ffind_wholeName%3DDichrostachys%2Bcinerea%26output_format%3Dnormal) | Fabaceae | Tree | Bark | Sore throat [71] | Alkaloids, coumarins, flavonoids, terpenoids [173] | Ethanol root extract showed activity against *P. aeruginosa* and *S. aureus* with MIC value of 5.5 mg/ml [174] |
| [*Dicoma capensis* Less.](http://www.ipni.org/ipni/idPlantNameSearch.do?id=201321-1&back_page=%2Fipni%2FeditSimplePlantNameSearch.do%3Ffind_wholeName%3DDicoma%2Bcapensis%2B%26output_format%3Dnormal) | Asteraceae | Herb | Leaves, roots, whole plant | Cold, influenza, asthma, cold, fever, TB [21,22,168] | Flavonoids [175] | - |
| *Dicoma anomala* Sond | Asteraceae | Herb | Tuber | Cough, TB [34,45] | Flavonoids [175] | Methanol and water roots extracts showed MICs >4 mg/ml against *S. aureus*, *S. pyogenes* and *P. aeruginosa* [176] |
| *Dicoma gerrardii* Harv. | Asteraceae | Herb | Roots | Cough [67] | - | - |
| *Dioscorea dregeana* T.Durand & Schinz. | Dioscoreaceae | Herb | Tuber | Sore throat [93,177] | Diosgenin [178] | Methanol tuber extract showed activity against *S. aureus* with MIC value of 0.81 mg ml [177] |
| ^#^*Dioscorea* *sylvatica* (Kunth) Eckl | Dioscoreaceae | Herb | Tuber | Chest pain [13] | Diosgenin [178] | Methanol tuber extract showed activity against *B. subtilis* with MIC value of 0.45 mg ml [177] |
| *Diosma acmaeophylla* Eckl. & Zeyh | Rutaceae | Shrub | Leaves | Cough, influenza [22] | - | - |
| *Diospyros austro-african* De Winter var austro-africana | Ebenaceae | Tree | Leaves | Cold [22] | - | - |
| *Diospyros mespiliformis* Hochst | Ebanaceae | Tree | Bark, leaves | TB, fever [66,132] | Alkaloids, glycosides, tannins, saponins [179] | Dichloromethane: methanol leaf extract showed activity against *P. aeruginosa* and *S. aureus* with MIC value of 1.00 mg/ml [180] |
| *Dittrichia graveolens* L. | Asteraceae | Herb | Whole plant | TB [95] | Essential oil [181] | Essential oil showed activity against *B. subtilis* with MIC value of 1 μl/ml [181]. Essential oil showed antioxidant activity using DPPH assay [181] |
| [*Dodonaea viscosa* (L.) Jacq.](http://www.ipni.org/ipni/idPlantNameSearch.do?id=53383-3&back_page=%2Fipni%2FeditSimplePlantNameSearch.do%3Ffind_wholeName%3DDodonaea%2Bangustifolia%2B%26output_format%3Dnormal) | Sapindaceae | Tree | Leaves | Chest complaints, cough, sore throat, cold, fever, lung infections, TB, influenza [15,16,22,1999] | Diterpenes, flavonoids, saponins [182] | Ethanol leaf extract showed activity against *K. pneumoniae*, *S. aureus* and *S. pyogens* with zone of inhibition ranging from 18 to 24 mm [183] |
| *Drimia depressa* (Baker) Jessop | Hyacinthaceae | Herb | Not specified | Chest pain, TB [34] | - | - |
| *Drosera capensis* L. | Droseraceae | Herb | Leaves | Fever, TB [184] | Flavonoids [185] | Ethanol leaf extract showed activity against *M. smegmatis* with MIC value of 3.12 mg/ml [186] |
| [*Ekebergia capensis* Sparrm.](http://www.ipni.org/ipni/idPlantNameSearch.do?id=578362-1&back_page=%2Fipni%2FeditSimplePlantNameSearch.do%3Ffind_wholeName%3DEkebergia%2Bcapensis%2B%26output_format%3Dnormal) | Meliaceae | Tree | Leaves | Chest pain, cough, runny nose [9,26] | Flavonoids, triterpenoids, steroids [187] | Water stem bark extract showed activity against *S. aureus* with 14.7 mm zone of inhibition [188] |
| [*Elephantorrhiza elephantina* (Burch.) Skeels](http://www.ipni.org/ipni/idPlantNameSearch.do?id=493740-1&back_page=%2Fipni%2FeditSimplePlantNameSearch.do%3Ffind_wholeName%3DElephantorrhiza%2Belephantina%2B%26output_format%3Dnormal) | Fabaceae | Shrub | Roots | Asthma, chest complaints, tonsillitis, TB [34,106,189, 190] | Flavonoids [191] | Methanol roots extract showed activity against S. aureus, K. pneumoniae, B. cereus with MIC values of 0.25 to 2.00 mg/ml [192] |
| [*Elytropappus rhinocerotis* Less.](http://www.ipni.org/ipni/idPlantNameSearch.do?id=203016-1&back_page=%2Fipni%2FeditSimplePlantNameSearch.do%3Ffind_wholeName%3DElytropappus%2Brhinocerotis%26output_format%3Dnormal) | Asteraceae | Shrub | Leaves | Chest complaints, sore throat, fever [16,40] | Flavonoids, labdane diterpene, rhinocerotinoic acid [193] | Methanol extract of aerial parts showed activity against *S. aureus* with 13 mm zone inhibition [194] |
| *Eriobotrya japonica* Lindl. | Rosaceae | Tree | Roots | TB [18] | Carotenoids, flavonoids, phenolic acids, phenolics, tannins, triterpenes [195] | Maslinic acid from leaves exhibited activity against *M. smegmatis*, *Mycobacterium phlei* and *M. tuberculosis* with zone of inhibition of 4.33 ± 0.33 to 8 ± 1.15 mm [196] |
| *Eriocephalus punctulatus* DC. | Asteraceae | Shrub | Leaves | Chest complaints [22] | Aliphatic esters, camphor, linalyl acetate, nerolidol, sesquiterpene lactones, spathulenol, terpenoid [197] | Methanol leaf extract showed activity against *B. subtilis* and *S. aureus* with MIC value of 0.3 to 0.4 mg/ml [78] |
| *Eriocephalus umbellulatus* Cass. | Asteraceae | Shrub | Whole plant | Chest complaints, cold [15] | - | - |
| [*Erythrina lysistemon* Hutch.](http://www.ipni.org/ipni/idPlantNameSearch.do?id=90039-3&back_page=%2Fipni%2FeditSimplePlantNameSearch.do%3Ffind_wholeName%3DErythrina%2Blysistemon%2B%26output_format%3Dnormal) | Fabaceae | Tree | Bark | Cold, fever [109] | Tetracyclic isoquinoline alkaloids [143] | Ethanol bark and leaf extract showed activities against *S. aureus* with 4 mm zone inhibition [198] |
| *Erythrina caffra* Thunb. | Fabaceae | Tree | Roots | Blocked nose, chest pain, fever [26] | Flavonoids [199] | Acetone bark extract showed activity against B. subtilis, K. pneumoniae and S. aureus with MIC value of 0.6250 mg/ml [200] |
| ^#^[*Erythrophleum* *lasianthum* Corbishley](http://www.ipni.org/ipni/idPlantNameSearch.do?id=494639-1&back_page=%2Fipni%2FeditSimplePlantNameSearch.do%3Ffind_wholeName%3DErythrophleum%2B%2Blasianthum%26output_format%3Dnormal) | Fabaceae | Tree | Leaves, stem | Bronchial infection, TB [5] | Alkaloids [201] | Methanol stem extract showed activity against *M. smegmatis* and *M. tuberculosis* with MIC value of 625 to 1250 μg/ml [5] |
| **Eucalyptus* *camadulensis* Dehnh | Myrtaceae | Tree | Bark, leaves, roots | Cough, TB [6,18] | Essential oils, cardiac glycosides, saponins, tannins [202,203] | Methanol leaf extract showed activity against *M. bovis* and *M. tuberculosis* with MIC value of 12.5 to 25 μg/ml [204] |
| *^ӿ^Eucalyptus* *globulus* Labill | Myrtaceae | Tree | Leaves | Chest complaints, cold, fever, influenza, cough [15,16] | Flavonoids, polyphenols [205] | Essential oil from leaves showed activity against *S. aureus* with 9 mm zone of inhibition [206] |
| **Eucalyptus* *grandis* W. Hill ex Maiden | Myrtaceae | Tree | Leaves | Chest pain, cold, cough, fever, runny nose, sore throat, tonsillitis [26] | Essential oil [207] | Essential oil from leaves showed activity against *B. cereus*, *P. aeruginosa*, *K. pneumoniae* and *S. aureus* with MIC value of 0.625 to 1.25 mg/ml [207] |
| *^ӿ^Eucalyptus sideroxylon* A. Cumm | Myrtaceae | Tree | Leaves | Cough, fever, influenza [22] | Polyphenols [208] | Essential oil from leaves showed activity against *B. cereus* and *S. aureus* with 16 to 17 mm zone of inhibition [208] |
| *Euclea divinorum* Hiern | Ebenaceae | Tree | Roots | Headache [132] | Amino acids, diterpenes, flavonoids, resins, tannins, triterpenoids [209] | Dichloromethane extract showed activity against *S. aureu* s and *B. subtilis* with 10± 0.41 to 10.75±1.6 mm zone of inhibition [209] |
| *Euclea natalensis* A.DC. | Ebenaceae | Tree | Bark, roots | Chest pain, fever, TB [39] | Naphthoquinones [210] | Ethanol root extract showed activity against *M. tuberculosis* at a concentration of 0.1 mg/mL [211] |
| [*Eucomis autumnalis* (Mill.) Chitt.](http://www.ipni.org/ipni/idPlantNameSearch.do?id=534988-1&back_page=%2Fipni%2FeditSimplePlantNameSearch.do%3Ffind_wholeName%3DEucomis%2Bautumnalis%26output_format%3Dnormal) | Hyacinthaceae | Herb | Bulb | Fever [67,143] | Eucomanalin, autumnariol, eucosterol [118, 212] | Acetone bulb extract showed activity against *B. subtilis* and *S. aureus* with MIC value of 0.78 mg/ml [213] |
| ^#^*Eucomis pallidiflora* [Baker subsp. pole-evansii (N.E.Br.) Reyneke ex J.C.Manning](http://redlist.sanbi.org/species.php?species=3790-22) | Hyacinthaceae | Herb | Bulb | Chest complaints, TB [17,18] | Homoisoflavonoids [214] | - |
| [*Eucomis schijffii* W.F.Reyneke](http://www.ipni.org/ipni/idPlantNameSearch.do?id=535006-1&back_page=%2Fipni%2FeditSimplePlantNameSearch.do%3Ffind_wholeName%3DEucomis%2Bschijffii%26output_format%3Dnormal) | Hyacinthaceae | Herb | Bulb | Cough [136] | Homoisoflavonoids, scillascillin [215] | Homoisoflavanones isolated from bulb showed activity against *S. aureus* with MIC values of 0.24 mg/ml [216] |
| *Euphorbia tirucalli* L. | Euphorbiaceae | Tree | Stem | Earache [26] | Alkaloids, essential oil, phenols, tannins [217] | Methanol stem extract showed activity against *S. aureus*, *S. pyogenes* and *K. pneumoniae* with 10 to 16 mm zone of inhibition [217] |
| [*Eulophia* *petersii* Rchb.f.](http://www.ipni.org/ipni/idPlantNameSearch.do?id=634845-1&back_page=%2Fipni%2FeditSimplePlantNameSearch.do%3Ffind_wholeName%3DEulophia%2Bpetersii%26output_format%3Dnormal) | Orchidaceae | Herb | Whole plant | Chest complaints [108] | Phenanthrenes, phytosterols [218] | Dichloromethane bulb extract showed activity against *B. subtilis, K*. *pneumoniae* and *S. aureus* with MIC value of 0.39 to 0.78 mg/ml [219] |
| [*Evolvulus* *alsinoides* Wall.](http://www.ipni.org/ipni/idPlantNameSearch.do?id=267956-1&back_page=%2Fipni%2FeditSimplePlantNameSearch.do%3Ffind_wholeName%3DEvolvulus%2Balsinoides%26output_format%3Dnormal) | Convolvulaceae | Herb | Leaves, stem | Cold, fever [132] | Alkaloids [220] | Methanol root extract showed activity against *S. aureus* and *B. cereus* with 14 to 17 mm zone of inhibition [220] |
| *Ficus burkei* Miq | Moraceae | Tree | Bark | Cold, throat infection [67] | - | - |
| **Ficus* *carica* L. | Moraceae | Tree | Bark | TB [18] | Anthocyanin, coumarins, organic acids, phenolic, phytosterols, triterpenoids [221] | Methanol leaf extract exhibited activities against *M. tuberculosis* with MIC value of 1600 µg/ml [222] |
| *Ficus sycomorus* L. | Moraceae | Tree | Bark, roots | Chest complaints, cold [132] | Alkaloids, flavonoids, saponins, setroids, tannins [223] | Acetone and methanol leaf extracts showed activities against *S. aureus* with MIC value of 6.6 to 8.7 mg/ml [223] |
| **Ficus* *platypoda* Miq. | Moraceae | Tree | Roots | TB [18] | Flavonoids, glycosides, sterols, tannins, triterpenes [224] | Petroleum ether leaf extract showed activity against *B. cereus* and *S. aureus* with 12 to 15 mm zone of inhibition [225] |
| *Ficus sur* Forssk. | Moraceae | Tree | Leaves | Ulceration of lung, TB [6] | Phenols, saponin glycosides, saponins, tannins, volatile oils [226] | Methanol stem extract showed activity against *B. cereus* *and S. aureus* with MIC value of 0.5 mg/ml [226] |
| ^ӿ^[*Foeniculum vulgare* Mill.](http://www.ipni.org/ipni/idPlantNameSearch.do?id=842680-1&back_page=%2Fipni%2FeditSimplePlantNameSearch.do%3Ffind_wholeName%3DFoeniculum%2Bvulgare%26output_format%3Dnormal) | Apiaceae | Herb | Leaves | Fever, chest pain, coughs, TB [39,40] | Essential oil, flavonoid, phenolic glycosides [227] | Essential oil extract from seeds showed activities against *B. subtilis* with MIC value of 0.25 mg/ml [228] |
| [*Galenia africana* L.](http://www.ipni.org/ipni/idPlantNameSearch.do?id=361862-1&back_page=%2Fipni%2FeditSimplePlantNameSearch.do%3Ffind_wholeName%3DGalenia%2Bafricana%2B%26output_format%3Dnormal) | Aizoaceae | Herb | Leaves | TB [184] | Alkaloids [229] | Ethanol leaf extract showed activity against *M. tuberculosis* with MIC value of 1.2 mg/ml [186] |
| [*Garuleum bipinnatum* Less.](http://www.ipni.org/ipni/idPlantNameSearch.do?id=208645-1&back_page=%2Fipni%2FeditSimplePlantNameSearch.do%3Ffind_wholeName%3DGaruleum%2Bbipinnatum%26output_format%3Dnormal) | Asteraceae | Herb | Roots | Chest complaint, fever, influenza [168] | Pimarane diterpenoid [230] | - |
| *Gerrardina foliosa* Oliv. | Flacourtiaceae | Tree | Bark, roots | Cough [189] | - | - |
| Geranium incanum Burm.f. | Geraniaceae | Herb | Leaves, stem | Chest complaints, sinusitis [16, 231] | Essential oils, flavonoids, saponins [232] | Water leaf extract inhibited growth of 83% of *K. pneumoniae* [233] |
| *Grewia villosa* Willd. var. villosa. | Malvaceae | Shrub | Roots | TB [66] | Alkaloids [234] | Water leaf extract showed activity against *B. cereus* and *S. aureus* with 16 to 22 mm zone of inhibition [235] |
| [*Gnidia cuneata* Meisn.](http://www.ipni.org/ipni/idPlantNameSearch.do?id=831713-1&back_page=%2Fipni%2FeditSimplePlantNameSearch.do%3Ffind_wholeName%3DGnidia%2Bcuneata%26output_format%3Dnormal) | Thymelaeaceae | Shrub | Leaves, stem | Fever [16] | - | - |
| *G*[*ymnosporia senegalensis* (Lam.) Loes](http://www.ipni.org/ipni/idPlantNameSearch.do?id=162084-1&back_page=%2Fipni%2FeditSimplePlantNameSearch.do%3Ffind_wholeName%3DMaytenus%2Bsenegalensis%26output_format%3Dnormal) | Celastraceae | Tree | Bark, roots | Pneumonia, TB [81] | Akaloids, monoterpenes, phenolic compounds, triterpenes [236] | Ethanol and hexane leaf extracts showed activities against *S. pneumoniae* and *S. aureus* with MIC values of 31.25 to 62.5 μg/ml [237] |
| Haemanthus albiflos L. | Amaryllidaceae | Herb | Bulb, leaves, roots | Cough, TB [6,13] | - | - |
| *Haemanthus coccineus* L. | Amaryllidaceae | Herb | Bulb | Chest complaints, TB [39] | Alkaloid narciclasine [238] | - |
| [*Haworthia limifolia* Marloth](http://www.ipni.org/ipni/idPlantNameSearch.do?id=536105-1&back_page=%2Fipni%2FeditSimplePlantNameSearch.do%3Ffind_wholeName%3DHaworthia%2Blimifolia%2B%26output_format%3Dnormal) | Xanthorrhoeaceae | Herb | Roots | Cough [239] | Alkaloids, flavonoids [164] | Ethyl acetate leaf extract showed activity against *B. subtilis*, *B. cereus* and *S. aereus* with MIC value of 3.0 to 4.0 mg/ml [240] |
| *Helichrysum caespititium* (DC.) Harv. | Asteraceae | Herb | Not specified | TB [34] | Caespitate [241] | Compound isolated from aerial parts showed activity against *B. cereus* and *S. aureus* with MIC values of 0.5 µg /ml [242] |
| [*Helichrysum crispum* D.Don](http://www.ipni.org/ipni/idPlantNameSearch.do?id=212626-1&back_page=%2Fipni%2FeditSimplePlantNameSearch.do%3Ffind_wholeName%3DHelichrysum%2Bcrispum%26output_format%3Dnormal) | Asteraceae | Herb | Leaves | Cold, cough, fever [40] | - | Acetone leaf extract showed activity against *B. subtilis*, *K. pneumoniae* and *S. aureus* with MIC value of 0.313 mg/ml [242] |
| [*Helichrysum herbaceum* Sweet](http://www.ipni.org/ipni/idPlantNameSearch.do?id=212848-1&back_page=%2Fipni%2FeditSimplePlantNameSearch.do%3Ffind_wholeName%3DHelichrysum%2Bherbaceum%26output_format%3Dnormal) | Asteraceae | Herb | Whole plant | Cough, cold, fever [81] | Flavones [243] | Acetone shoot extract showed activity against B. subtilis, B. cereus and S. aereus with MIC value of 1.0 mg/ml [242] |
| [*Helichrysum kraussii* Sch.Bip.](http://www.ipni.org/ipni/idPlantNameSearch.do?id=212914-1&back_page=%2Fipni%2FeditSimplePlantNameSearch.do%3Ffind_wholeName%3DHelichrysum%2Bkraussii%2B%26output_format%3Dnormal) | Asteraceae | Herb | Leaves | Cough, TB, blocked nose, chest pain [26,136] | Terpenoids [244] | Acetone shoots extracts is effective against *B. subtilis*, *B. cereus* and *S. aereus* with MIC of 1.0 (mg/ml) [242] |
| [*Helichrysum nudifolium* Less.](http://www.ipni.org/ipni/idPlantNameSearch.do?id=213099-1&back_page=%2Fipni%2FeditSimplePlantNameSearch.do%3Ffind_wholeName%3DHelichrysum%2Bnudifolium%2B%26output_format%3Dnormal) | Asteraceae | Herb | Roots, leaves | Cold, cough, chest complaints [15,81] | Diterpenes [245] (Seaman et al., 2012) | Acetone shoot extract showed activity against *B. subtilis*, *B. cereus* and *S. aereus* with MIC value of 1.0 mg/ml [242] |
| [*Helichrysum odoratissimum* Sweet](http://www.ipni.org/ipni/idPlantNameSearch.do?id=213115-1&back_page=%2Fipni%2FeditSimplePlantNameSearch.do%3Ffind_wholeName%3DHelichrysum%2Bodoratissimum%26output_format%3Dnormal) | Asteraceae | Herb | Leaves, roots, whole plant | Chest pain, flu [13,190,231] | Monoterpenes, diterpenes 242] | Acetone shoot extract showed activity against *B. subtilis*, *B. cereus* and *S. aereus* with MIC value of 1.0 mg/ml [242] |
| [*Helichrysum ruderale* Hilliard & B.L.Burtt](http://www.ipni.org/ipni/idPlantNameSearch.do?id=213272-1&back_page=%2Fipni%2FeditSimplePlantNameSearch.do%3Ffind_wholeName%3DHelichrysum%2Bruderale%2B%26output_format%3Dnormal) | Asteraceae | Herb | Whole plant | Cough, cold, fever [81] | Diterpenes [245] | Chloroform: methanol extract showed activity against *B. cereus* and *S. aereus* with MIC value of 1.0 to 2 mg/ml [242] |
| [*Helichrysum simillimum* DC.](http://www.ipni.org/ipni/idPlantNameSearch.do?id=213334-1&back_page=%2Fipni%2FeditSimplePlantNameSearch.do%3Ffind_wholeName%3DHelichrysum%2Bsimillimum%2B%26output_format%3Dnormal) | Asteraceae | Herb | Leaves | Cough, cold, fever [81] | Flavonol kaempferol [246] | Acetone shoot extract showed activity against *B. subtilis*, *B. cereus* and *S. aereus* with MIC value of 1.0 mg/ml [242] |
| [*Helichrysum umbraculigerum* Less.](http://www.ipni.org/ipni/idPlantNameSearch.do?id=213451-1&back_page=%2Fipni%2FeditSimplePlantNameSearch.do%3Ffind_wholeName%3DHelichrysum%2Bumbraculigerum%2B%26output_format%3Dnormal) | Asteraceae | Herb | Whole plant | Cough, cold, fever [81] | Resorcinol [247] | Acetone shoot extract showed activity against *B. subtilis*, *B. cereus* and *S. aereus* with MIC value of 1.0 mg/ml [242] |
| [*Hermbstaedtia glauca* Rchb. ex Steud.](http://www.ipni.org/ipni/idPlantNameSearch.do?id=60690-1&back_page=%2Fipni%2FeditSimplePlantNameSearch.do%3Ffind_wholeName%3DHermbstaedtia%2Bglauca%2B%26output_format%3Dnormal) | Amaranthaceae | Shrub | Whole plant | Fever [22] | - | - |
| [*Hermannia cuneifolia* Jacq.](http://www.ipni.org/ipni/idPlantNameSearch.do?id=823680-1&back_page=%2Fipni%2FeditSimplePlantNameSearch.do%3Ffind_wholeName%3DHermannia%2Bcuneifolia%26output_format%3Dnormal) | Sterculiaceae | Shrub | Leaves | Influenza, sore throat [168] | β-sitosterol and lupeol [248] | Acetone extract of whole plant showed activity against *C. neoformans* with MIC values of 0.5 mg/ml [248] |
| [*Hermannia depressa* N.E.Br.](http://www.ipni.org/ipni/idPlantNameSearch.do?id=823688-1&back_page=%2Fipni%2FeditSimplePlantNameSearch.do%3Ffind_wholeName%3DHermannia%2Bdepressa%2B%26output_format%3Dnormal) | Sterculiaceae | Herb | Bark | Fever [108] | Saponins, tannins [249] | Ethyl acetate leaf extract showed activity against *B. subtilis*, *K. pneumoniae* and *S. aureus* with MIC value of 0.78 to 1.56 mg/ml [249] |
| [*Heteropyxis natalensis* Harv.](http://www.ipni.org/ipni/idPlantNameSearch.do?id=553494-1&back_page=%2Fipni%2FeditSimplePlantNameSearch.do%3Ffind_wholeName%3DHeteropyxis%2Bnatalensis%26output_format%3Dnormal) | Myrtaceae | Tree | Roots | Unspecified respiratory infections [136] | Monoterpenes [250] | Oil extracted from aerial parts showed activity against *C. neoformans* with MIC values of 2.0 to 3.0 mg/ml [251] |
| *Hippobromus pauciflorus* (L.f.) Radlk. | Sapindaceae | Tree | Bark | Cough, TB [6,13] | Cardiac glycosides, flavonoids, saponins, steroids, tannins, terpenes [100] | Acetone root extract showed activity against *B. cereus*, *K. pneumoniae*, *S. aureus* and *S. epidermidis* with MIC value of 0.5 to 10 mg/ml [100] |
| *Hypoxis argentea* Harv. ex Baker | Hypoxidaceae | Herb | Leaves | TB [6] | - | - |
| *Hypoxis hemerocallidea* Fisch., C.A.Mey. & Avé-Lall. | Hypoxidaceae | Herb | Tuber | TB [18,33] | β-sitosterol, diglucoside hypoxoside, aglycone rooperol, sterols, sterolins [252] | Ethyl acetate corm extract exhibited activities against *S. aureus* with MIC value of 0.31 mg/ml [252] |
| *Hypoxis cf. acuminata* Baker | Hypoxidaceae | Herb | Tuber | Blocked and runny nose, chest pain, cold, fever [26] | Geraniol acuminoside, hypoxoside [252] | Dichloromethane: methanol tuber extract showed activity against *C. neoformans* with MIC value of 0.87 mg/ml [26] |
| *Jamesbrittenia atropurpurpea* (Benth.) Hilliard | Scrophulariaceae | Shrub | Whole plant | Bronchitis, cough [15] | - | - |
| *Kedrostis africana* (L.) Cogn. | Curcubitaceae | Herb | Leaves, roots | Bronchitis, cough, cold, fever [13] | Alkaloids, saponin [253] | - |
| *Krauseola mosambicina* (Moss) Pax & K.Hoffm. | Caryophyllaceae | Herb | Stem, leaves | Blocked and runny nose, cough [26] | - | Dichloromethane: methanol leaf and stem extract showed activity against *C. neoformans* with MIC value of 1.0 mg/ml [26] |
| [*Lantana rugosa* Thunb.](http://www.ipni.org/ipni/idPlantNameSearch.do?id=863429-1&back_page=%2Fipni%2FeditSimplePlantNameSearch.do%3Ffind_wholeName%3DLantana%2Brugosa%26output_format%3Dnormal) | Verbenaceae | Shrub | Leaves | Bronchial infection, fever [51,132] | Alkaloid, hydrocarbon, lantanin, volatile oil [1,136] | Acetone leaf extract showed activity against *P. aeruginosa* and *S. aureus* with MIC values of 0.39 to 1.6 mg/ml [254] |
| *^ӿ^Lantana camara* L. | Verbenaceae | Shrub | Leaves | Fever, flu [255] | Alkaloids, essential oils, flavonoids, glycosides, phenolic compounds, quinine, saponins, steroids, triterpens [256] | Ethyl acetate root bark extract showed activity against *B. subtilis* and *S. aureus* with 32 to 40 mm zone of inhibition [257] |
| [*Leonotis intermedia* Lindl.](http://www.ipni.org/ipni/idPlantNameSearch.do?id=449127-1&back_page=%2Fipni%2FeditSimplePlantNameSearch.do%3Ffind_wholeName%3DLeonotis%2Bintermedia%26output_format%3Dnormal) | Lamiaceae | Shrub | Leaves | Chest complaints, cold, influenza, bronchitis, TB [168] | Flavonoid, iridoid, phenolic [258] | Ethanol leaf extract showed activity against *M. aurum* and *S. aureus* with MIC value of 0.195 to 0.78 mg/ml [76] |
| *Leonotis lanceolata* L. | Fabaceae | Shrub | Not specified | Cough, TB [34] | - | - |
| [[*Leonotis leonurus* (L.) R.Br.](http://www.ipni.org/ipni/idPlantNameSearch.do?id=449133-1&back_page=%2Fipni%2FeditSimplePlantNameSearch.do%3Ffind_wholeName%3DLeonotis%2Bleonurus%26output_format%3Dnormal)](http://www.ipni.org/ipni/idPlantNameSearch.do?id=449133-1&back_page=%2Fipni%2FeditSimplePlantNameSearch.do%3Ffind_wholeName%3DLeonotis%2Bleonurus%26output_format%3Dnormal) | Lamiaceae | Shrub | Leaves, stem, roots | Cough, cold, influenza, bronchial infection [1] | Flavonoids, phenolics, terpenes [70] | Methanol leaf extract showed activity against *M. smegmatis* with MIC value of 15.0 mg/ml [70] |
| [*Leonotis ocymifolia* (Burm.f.) Iwarsson](http://www.ipni.org/ipni/idPlantNameSearch.do?id=915192-1&back_page=%2Fipni%2FeditSimplePlantNameSearch.do%3Ffind_wholeName%3DLeonotis%2Bocymifolia%26output_format%3Dnormal) | Lamiaceae | Shrub | Leaves, stem | Asthma, TB [16,168] | Diterpenes, leonitin [259] | Methanol leaf extract showed activity against *K. pneumoniae* and *S. aureus* with 14.7 to 15.0 zone of inhibition [260] |
| *Lepidium capense* Thunb. | Boraginaceae | Herb | Tuber | Cough [9] | - | Water leaf extract showed antioxidant activity using DPPH method [261] |
| *Leysera gnaphalodes* (L.) L. | Asteraceae | Shrub | Whole plant | Catarrh, cough, TB [15,95] | Oleanolic acid pentacyclic, triterpenoids [95] | Compound from entire plant showed activity against *Mycobacterium avium* and *Mycobacterium scrofulaceum* with MIC value of 1.25 mg/ml [95] |
| *Lichtensteina lacera* Cham. & Schltdl. | Apiaceae | Shrub | Bulb, leaves, whole plant | Cough, asthma, fever, chest complaints [16,231] | - | - |
| *Lippia javanica* (Burm.f.) Spreng. | Verbenaceae | Shrub | Leaves | Cough, flu, cold, blocked runny nose, chest pain, fever, sore throat, tonsillitis, TB [18,26,66,132] | Triterpenoid carboxylic acid [262] | Dichloromethane: methanol leaf extract showed activity against *C. neoformans* with MIC value of 0.25 mg/ml [26]. Triterpenoid carboxylic acid and euscaphic acid isolated from leaves showed activity against *M. tuberculosis* with MIC value of 50 μg/ml [262] |
| *Lonchocarpus capassa* Rolfe | Fabaceae | Tree | Bark | Cold [263] | Lectin [264] | Chloroform leaf extract showed activity against *K. pneumoniae* and *P. aeruginosa* with MIC value of 3.125 mg/ml [265] |
| *Loxostylis alata* Spreng.f.ex Reichb. | Anacardiaceae | Tree | Bark | Asthma, chest complaints and cough [16] | Terpenes [266] | Acetone leaves extract is effective against *S. aureus* and *P. aeruginosa* with MIC value of 0.16 (mg/ml) using microdilution method [267] |
| *Lycium ferocissimum* Miers | Solanaceae | Shrub | Whole plant | Asthma, chest complaints [16] | — | — |
| *Maerua angolensis* DC. | Capparaceae | Tree | Leaves | Headache [132] | Alkaloids, cardiac glycosides, flavonoids, tannins [268] | Methanol leaf extract showed activity against *B. subtilis*, *K. pneumoniae*, *P. aeruginosa*, *S.* *aureus* and *S. pyogenes* with MIC value of 6.25 to 25 mg/ml [268] |
| *^ӿ^Malva parviflora* L. | Malvaceae | Herb | Leaves | Sore throat [15] | Alkaloids, flavonoids, tannins, phenols, saponins [269] | Methanol seed extract showed activity against *Streptococcus pneumoniae* and *S. aureus* with 13 to 19 mm zone of inhibition [270] |
| *Melolobium obcordatum* Harv. | Fabaceae | Herb | Not specified | Cough, sore throat, TB [34] | - | - |
| *Mentha longifolia* L. | Lamiaceae | Herb | Leaves | Asthma, cold, fever, cough, ulceration of the lung [6,16,33,40] | Flavonoids, tannins, saponins [271] | Ethyl acetate leaf extract showed activity against *K. pneumoniae*, *P. aeruginosa* and *S. aureus* with MIC value of 1.25 mg/ml [272] |
| *^ӿ^Mentha spicata* L. | Lamiaceae | Herb | Leaves | Cold [168] | Essential oil [273] | Essential oil showed activity against *B. cereus*, *B. subtilis* and *S. aureus* with 10 to 18 mm zone of inhibition [273] |
| *Mentha spp*. | Lamiaceae | Herb | Leaves | TB [18] | - | - |
| ^#^*Merwilla* *plumbea* (Lindl.) Speta | Hyacinthaceae | Herb | Bulb | Chest complaints, TB, influenza [17,18,35] | Flavonoids, saponins, tannin [274] | Ethanol bulb extract showed activity against *B. subtilis*, *K. pneumoniae* and *S. aureus* with MIC value of 3.13 mg/ml [275] |
| *Monsonia burkeana* Planch. ex Harv. | Geraniaceae | Herb | Whole plant | Chest complaints, cough [15] | Polyphenols, tannins [276] | Ethanol leaf extract showed activity against *B. cereus* and *K. pneumoniae*, *S. aureus* with MIC value of 3.13 mg/ml [276] |
| *Myrica serrata* Lam. | Myricaceae | Tree | Bark, roots | Cough [49] | Cryptostrobin, flavanones demethoxymatteucinol [277] | Dichloromethane leaf extract showed activity against *B. subtilis* [277] |
| *Myrothamnus flabellifolius* (Sond.) Welw. | Myrothamnaceae | Herb | Whole plant | Cold, chest complains, flu, TB [1,18,132] | Alkaloids, disaccharide, essential oils, flavonoids, phenolics, polyphenol, tannins [278] | Essential oils showed activity against *C. neoformans* and *S. aureus* with 12 to 22 mm zone of inhibition [279] |
| #*Notobubon pearsonnii* (Adamson) Magee | Apiaceae | Shrub | Leaves | Asthma, pneumonia, TB [22] | - | - |
| [*Nymphaea nouchali* Burm.f.](http://www.ipni.org/ipni/idPlantNameSearch.do?id=605643-1&back_page=%2Fipni%2FeditSimplePlantNameSearch.do%3Ffind_wholeName%3DNymphaea%2Bnouchali%2B%26output_format%3Dnormal) | Nymphaeaceae | Herb | Roots | Cold, cough [1] | Alkaloid, flavonoid, tannin, terpinoid [280] | Chloroform, ethanol and hexane leaf extracts showed activities *against B. subtilis*, *P. aeruginosa*, *S. aureus* and *Streptococcus mutans* with 7.1 to 12.6 mm zone of inhibition [280] |
| [*Ocimum basilicum* L.](http://www.ipni.org/ipni/idPlantNameSearch.do?id=108348-3&back_page=%2Fipni%2FeditSimplePlantNameSearch.do%3Ffind_wholeName%3DOcimum%2Bbasilicum%26output_format%3Dnormal) | Lamiaceae | Herb | Leaves, stem | Chest complaints [231] | Cardiac glycosides, essential oil, phenolic compounds, saponins, tannins [281] | Methanol leaf extract showed activity against *P. aeruginosa* and *S.* *aureus* with 13 to 15 mm zone of inhibition [282] |
| *Olea capensis* L. | Oleaceae | Tree | Leaves | Sore throat, TB [95] | - | - |
| *Olea europaea* L. | Oleaceae | Tree | Leaves | Chest pain, bronchitis [22] | Biophenols, flavanones, flavone glycosides, flavonoids, iridane glycosides, secoiridoids, iridoids, secoiridoid glycosides, triterpenes [283] | Aqueous leaf extract showed activity against *K. pneumoniae*, *P. aeruginosa* and *S. aureus* with 9.0 to 13.3 mm zone of inhibition [283] |
| *Oncosiphon piluliferum* (L.f.) Källersjö | Asteraceae | Herb | Whole plant | Fever, influenza [15] | Sesquiterpene lactones [284] | - |
| [*Osmitopsis asteriscoides* Cass.](http://www.ipni.org/ipni/idPlantNameSearch.do?id=235744-1&back_page=%2Fipni%2FeditSimplePlantNameSearch.do%3Ffind_wholeName%3DOsmitopsis%2Basteriscoides%26output_format%3Dnormal) | Asteraceae | Herb | Leaves | Chest complaints, cough [15] | Essential oil, sesquiterpene lactones [285] | Essential oil showed activity against *B. subtilis*, *C. neoformans*, *P. aeruginosa* and *S. aureus* with MIC value of 8.0 to 32.0 mg/ml [285] |
| [*Osteospermum imbricatum* L.](http://www.ipni.org/ipni/idPlantNameSearch.do?id=235819-1&back_page=%2Fipni%2FeditSimplePlantNameSearch.do%3Ffind_wholeName%3DOsteospermum%2Bimbricatum%26output_format%3Dnormal) | Asteraceae | Shrub | Bulb, leaves, roots | Chest complaints [16,231] | - | - |
| *Ozoroa obovata* (Oliv) R.Fern. & A.Fern. | Anacardiaceae | Tree | Leaves | Blocked and runny nose, cough, fever [26] | - | Dichloromethane: methanol leaf extract showed activity against *C. neoformans* with MIC value of 0.83 mg/ml [26] |
| *Parapodium costatum* E.Mey. | Apocynaceae | Herb | Not specified | Short of breath, TB [34] | - | - |
| *Parinari capensis* Harv. subsp. incohata F. White | Chrysobalanaceae | Shrub | Roots | TB [26] | Diterpene lactones [286] | Dichloromethane: methanol leaf extract showed activities against *C. neoformans a*nd *M. catarrhalis* with MIC value of 0.03 to 0.50 mg/ml [26] |
| *Pentanisia prunelloides* (Klotzsch ex Eckl. & Zeyh.) Walp | Rubiaceae | Herb | Tuber | Chest pain, short of breath, TB [13,34] | Cardiac glycosides, tannin [287] | Ethanol leaf extract showed activity against *K. pneumoniae*, *M. aurum*, *M. tuberculosis* and *S. aureus* with MIC value of 0.39 to 0.78 mg/ml [76] |
| [*Pelargonium abrotanifolium* (L.f.) Jacq.](http://www.ipni.org/ipni/idPlantNameSearch.do?id=375560-1&back_page=%2Fipni%2FeditSimplePlantNameSearch.do%3Ffind_wholeName%3DPelargonium%2Babrotanifolium%2B%26output_format%3Dnormal) | Geraniaceae | Shrub | Leaves | Chest complaints, cold, influenza [21] | - | - |
| [*Pelargonium grossularioides* (L.) L'Hér.](http://www.ipni.org/ipni/idPlantNameSearch.do?id=376265-1&back_page=%2Fipni%2FeditSimplePlantNameSearch.do%3Ffind_wholeName%3DPelargonium%2Bgrossularioides%2B%26output_format%3Dnormal) | Geraniaceae | Herb | Whole plant | Asthma, chest complaints [21] | Tannins, saponins [288] | Some activity was recorded against *S. aureus* and *M. smegmatis* [288] |
| *Pelargonium myrrhifolium* (L.) L’Hér. | Geraniaceae | Herb | Roots | TB [15] | - | - |
| *Pelargonium ramosissimum* (Cav.) Willd. | Geraniaceae | Herb | Whole plant | Cold, TB [15] | - | - |
| *Pelargonium reniforme* Curtis | Geraniaceae | Herb | Tuber | Cough, TB [289] | Coumarins, scopoletin [290] | Acetone, chloroform and ethanol root extracts showed activity against *M. tuberculosis* with MIC value of 10.3 mg/ml [290] |
| *Pelargonium sidioides* DC. | Geraniaceae | Herb | Not specified | Chest pains, cough, TB [34] | Alkaloides, essential oils, flavonoids, peptides [291] | Water leaf extract showed activity against *S. pyogenes*, *K. pneumoniae* and *S. aureus* with MIC values of 0.78 mg/ml [80] |
| *Pellaea calomelanos* (Sw.) Link | Pteridaceae | Herb | Roots | TB [18] | Triterpenoid saponins [118] | Aerial parts extract showed activity against *S. aureus*, *B. cereus*, *K. pneumoniae* and *C. neoformans* with MIC values of 0.53 to 1.00 mg/ml [292] |
| *Peltophorum africanum* Sond | Fabaceae | Tree | Bark | Chest complaints, fever, sore throat, TB [6,132] | Benzenoids, coumarins, flavanols, terpenes, xanthone [293] | Bark and root extracts showed activities against *K. pneumoniae*, *P. aeruginosa*, *S. aureus* and S*. pyogenes* with MIC values of 0.08 to 6 mg/ml [293] |
| [*Pegolettia baccharidifolia* Less.](http://www.ipni.org/ipni/idPlantNameSearch.do?id=236730-1&back_page=%2Fipni%2FeditSimplePlantNameSearch.do%3Ffind_wholeName%3DPegolettia%2Bbaccharidifolia%26output_format%3Dnormal) | Asteraceae | Shrub | Twigs | Asthma, breathing problems [168] | - | - |
| [*Pentzia incana* Druce](http://www.ipni.org/ipni/idPlantNameSearch.do?id=236838-1&back_page=%2Fipni%2FeditSimplePlantNameSearch.do%3Ffind_wholeName%3DPentzia%2Bincana%2B%26output_format%3Dnormal) | Asteraceae | Shrub | Twigs | Cold, flu [168] | - | Ethanol leaf and stem extract showed activities against *S. aureus* with MIC value of 166.7 mg/ml [294] |
| [*Pentzia punctata* Harv.](http://www.ipni.org/ipni/idPlantNameSearch.do?id=236850-1&back_page=%2Fipni%2FeditSimplePlantNameSearch.do%3Ffind_wholeName%3DPentzia%2Bpunctata%26output_format%3Dnormal) | Asteraceae | Shrub | Not specified | Cold ([168] | - | - |
| [*Petroselinum crispum* (Mill.) Fuss](http://www.ipni.org/ipni/idPlantNameSearch.do?id=60442790-2&back_page=%2Fipni%2FeditSimplePlantNameSearch.do%3Ffind_wholeName%3DPetroselinum%2Bcrispum%26output_format%3Dnormal) | Apiaceae | Herb | Leaves | Cough [40] | Alkaloids, flavonoids, phenolic copounds, saponins, tannins [295] | Hydro alcohol and petroleum ether leaf extracts showed activities against *S. aureus*, *K. pneumoniae* and P. aeruginosa with 12 to 20 mm zone of inhibition [295] |
| *Pharnaceum lineare* L.f. | Molluginaceae | Herb | Whole plant | TB [15] | - | - |
| *Phyllanthus meyerianus* Müll. Arg. | Phyllanthaceae | Tree | Bark, roots | Cough [49] | - | - |
| *Phyllanthus reticulatus* Lodd. | Phyllanthaceae | Tree | Leaves | Sore throat [1] | Betulin, epifriedelinol, taraxerone, taraxeryl acetate [296] | Ethanol leaf extract showed activities against *S. aureus* and *P. aeruginosa* with 16.7 to 18.3 zone of inhibition [297] |
| *Piper capense* L.f. | Piperaceae | Shrub | Roots | Chest complaints, sore throat, TB [66,71] | Alkaloids, chalcones, dihydrochalcones, flavanones, flavones, neolignans, piperolides, propenylphenols, steroids, terpenes [298] | Acetone root extract showed activity against *M. tuberculosis* with MIC value of 50 mg/ml [66] |
| *Pittosporum viridiflorum* Sims | Pittosporaceae | Shrub | Bark | Asthma, lung infections, chest complaints, fever [13,16,33] | Flavonoids, phenolic[299] | Ethanol leaf extract showed activity against *B. cereus* with 1.7 mm zone of inhibition [300] |
| *Pteronia camphorate* L. | Asteraceae | Herb | Leaves | TB [22] | - | - |
| *Plectostachys serpyllifolia* (P.J.Bergius) Hilliard & B.L.Burtt | Asteraceae | Herb | Leaves | Chest complaints, cold [15] | Essential oil [301] | Methanol leaf extract showed activity against *B. subtilis*, *P. aeruginosa*, *S. aureus* and *S. epidermis* with MIC value of 5.0 mg/ml [301] |
| *Plectranthus neochilus* Schltr. | Lamiaceae | Herb | Leaves | Blocked and runny nose, chills, cough [26] | Essential oil [302] | Essential oil extracted from leaves showed activity against against *Streptococcus salivarus*, *Streptococcus sobrinus*, *Streptococcus sanguinis*, *Streptococcus mitis* and *S. mutans* with MIC values of 31.3 to 250.0 𝜇g/ml [302] |
| [*Pouzolzia mixta* Solms](http://www.ipni.org/ipni/idPlantNameSearch.do?id=856187-1&back_page=%2Fipni%2FeditSimplePlantNameSearch.do%3Ffind_wholeName%3DPouzolzia%2Bmixta%26output_format%3Dnormal) | Urticaceae | Shrub | Bark | Fever [81] | - | Methanol leaf extract showed activity against *S. aureus*, *S. epidermis* and *B. subtilis* with MIC value of 0.28 to 0.58 mg/ml [303] |
| *Priva cordifolia* Druce | Verbenaceae | Herb | Seeds | Sore throat [1] | - | - |
| ^#^ ^α^ *Prunus* *africana* Hook. | Rosaceae | Tree | Bark, leaves | Chest pain, cough, fever, TB [6, 184] | Campesterol, β-sitosterol, lup-20(29)-en-3-one, palmitic acid, β-sitostenone [304] | Methanol stem bark extract showed activity against *S. aureus*, *S. pneumoniae* with MIC values of 0.073 to 2.5 mg/ml [305] |
| **Psidium guajava* L. | Myrtaceae | Tree | Leaves | Blocked and runny nose, chills, cough, fever, sore throat [26] | Alkaloids, carbohydrates, flavonoids, saponins, sterols, tannins, triterpenoids [306] | Dichloromethane: methanol leaf extract showed activities against *C. neoformans* and *S. aureus* with MIC value of 0.50 mg/ml [26] |
| *Ptaeroxylon obliquum* Thumb | Rutaceae | Tree | Bark, leaves | Sinusitis, chest complaints [6,16] | Phenolic compounds [307] | Ethanol leaf extract showed activity against S. aureus and S. pneumoniae with MIC value of 32 mg/ml [308] |
| [*Pteronia incana* DC.](http://www.ipni.org/ipni/idPlantNameSearch.do?id=240266-1&back_page=%2Fipni%2FeditSimplePlantNameSearch.do%3Ffind_wholeName%3DPteronia%2Bincana%26output_format%3Dnormal) | Asteraceae | Shrub | Leaves | Cough [106] | Essential oil [309] | Methanol: dichloromethane extracts from essential oil showed activity against *M. smegmatis*, *C. neoformans*, *M. catarrhalis* and *K. pneumoniae* with MIC values of 0.3 to 1.3 mg/ml [309] |
| *Rafnia amplexicaulis* (L.) Thunb. | Fabaceae | Tree | Whole plant, roots | Asthma, influenza, cough [15,16] | Quinolizidine alkaloids [310] | - |
| *Ranunculus multifidus* Forssk. | Ranunculaceae | Herb | Leaves | Cough [9] | Alkaloids, flavonoid, saponins,steroids, tannins [311] | Petroleum ether stem extract showed activity against *S. aureus* and *P. aeruginosa* with MIC value of 5.6 mg/ml [311] |
| [*Rapanea melanophloeos* Mez](http://www.ipni.org/ipni/idPlantNameSearch.do?id=589728-1&back_page=%2Fipni%2FeditSimplePlantNameSearch.do%3Ffind_wholeName%3DRapanea%2Bmelanophloeos%26output_format%3Dnormal) | Myrsinaceae | Tree | Bark | Sore throat [132] | Benzoquinones, flavonoids, saponins, tannins, terpenes, triterpenoid [312] | Acetone leaf extract showed activity against *M. smegmatis*, *M. aurum*, M*. tuberculosis* with MIC value of 0.156 to 0.625 mg/ml [313] |
| [*Rauvolfia caffra* Sond.](http://www.ipni.org/ipni/idPlantNameSearch.do?id=81519-1&back_page=%2Fipni%2FeditSimplePlantNameSearch.do%3Ffind_wholeName%3DRauvolfia%2Bcaffra%26output_format%3Dnormal) | Apocynaceae | Tree | Bark | Fever [118] | Alkaloid [314] | Alkaloid extract from roots showed activity against Mycobacterium madagascariense and Mycobacterium indicus pranii with MIC value of 1.25 μg/ml [314] |
| [*Rhamnus prinoides* L'Hér.](http://www.ipni.org/ipni/idPlantNameSearch.do?id=718580-1&back_page=%2Fipni%2FeditSimplePlantNameSearch.do%3Ffind_wholeName%3DRhamnus%2Bprinoides%2B%26output_format%3Dnormal) | Rhamnaceae | Tree | Leaves | Pneumonia [81] | Alkaloids, flavonoids, polyphenols, saponins, tannins, terpenoids [315] | Acetone leaf extract showed activity against *M. smegmatis*, *M. tuberculosis*, *P. aeruginosa*, *S. pneumonia*, *S. aureus* and *S. pyogen* with MIC value of 0.625 to 8.13 mg/ml [313, 315] |
| *Searsia lancea* (L.f.) F.A. Barkley | Anacardiaceae | Tree | Leaves | Cold, fever [132] | Essential oil [316] | Essential oil showed activity against *B. subtilis*, *K. pneumoniae*, *P. aeruginosa* and *S. aureus* with 4.1 to 9.0 mm zone of inhibition [316] |
| *Rhus rogersii* Schönland | Anacardiaceae | Tree | Bark | TB [66] | - | Acetone bark extract showed against *M. tuberculosis* with MIC value of 50 mg/ml [66] |
| *Rhipsalis baccifera* (J.Mill.) Stearn subsp. mauritiana (DC.) Barthlott | Cactaceae | Herb | Stem, roots | Chest complaints [16] | - | - |
| *Rhoicissus tridentata* Wild and Drum | Vitaceae | Tree | Leaves, tuber | TB [66] | Catechin, epicatechin, epigallocatechin gallate, gallic acid [317] | Acetone bark extract showed activity against M. tuberculosis with MIC value of 50 mg/ml [66] |
| ^ӿ^*Ricinus communis* L. | Euphorbiaceae | Shrub | Bark, leaves, seeds | Asthma, fever, flu, sore throat, TB [1,318] | Alkaloids, flavonoids, glycosides, saponins, sterols, tannins, terpenoids [1,319] | Methanol leaf extract showed activity against *B. subtilis*, *S. aureus* and *K. pneumonia* with 16.3 to 20.7 mm zone of inhibition [320] |
| **Rorippa nasturtium*-aquaticum (L.) Hayek | Brassicaceae | Herb | Leaves | Bronchitis, lung infections [15] | - | Ethanol whole plant extract showed activity against *S. aureus* with 7 mm zone of inhibition [321] |
| **Rosmarinus officinalis* L. | Lamiaceae | Herb | Leaves | Asthma, chest problems, TB [6, 15] | Camphor, limonene, terpineol, terpinolene [322] | LeaF extract showed activity against P. aeruginosa, S. aureus and B. cereus with MIC value of 0.06 to 1.0 mg/ml [322] |
| *Rubia petiolaris* DC. | Rubiaceae | Shrub | Leaves | TB [6] | - | - |
| [*Rumex crispus* L.](http://www.ipni.org/ipni/idPlantNameSearch.do?id=224413-2&back_page=%2Fipni%2FeditSimplePlantNameSearch.do%3Ffind_wholeName%3DRumex%2Bcrispus%2B%26output_format%3Dnormal) | Polygonaceae | Herb | Leaves | Cough [54] | Phenolics [323] | Methanol aerial part extract showed activity against B. cereus and B. subtilis with MIC values of 31.3 to 125 μg/ml [323] |
| *Rumex lanceolatis* Thunb. | Polygonaceae | Herb | Not specified | Flu, sore throat, TB [34] | - | - |
| ^ӿ^[*Ruta graveolens* L.](http://www.ipni.org/ipni/idPlantNameSearch.do?id=775099-1&back_page=%2Fipni%2FeditSimplePlantNameSearch.do%3Ffind_wholeName%3DRuta%2Bgraveolens%2B%26output_format%3Dnormal) | Rutaceae | Herb | Leaves | Chest pain, fever, cold, influenza [15,16,21] | Rutin, rutamarin, furanocoumarins, quinolinic alkaloids [324] | Ethanol leaf extract showed activity against *S. aureus* and *B. subtilis* with 19.4 ± 0.24 to 22.0 ± 0.04 mm zone of inhibition [325] |
| *^ӿ^Salix babylonica* L. | Salicaceae | Tree | Bark | Fever [168] | Flavonoid, salicylate, trichocarpin [326] | - |
| *Salix mucronata* Thunb. | Salicaceae | Tree | Bark, seeds | Fever, TB [15,18] | Alkaloids, cardiac glycosides, flavonoids, phenols, saponins, sterols, tannins, triterpenoids [327] | Ethanol leaf extract showed activity against *M. aurum* with MIC value of 1.56 mg/ml [328] |
| *Salvia africana-caerulea* L. | Lamiaceae | Shrub | Leaves | Cough, cold [168] | Alkaloids, essential oils [329] (Kamatou, 2006) | Methanol :chloroform aerial parts extract showed activity against K. *pneumoniae*, *B. cereus*, *S. aureus* and *M. tuberculosis* with MIC values of 0.5 to 6.0 mg/ml [329] |
| *Salvia africana-lutea* L. | [Lamiaceae](http://en.wikipedia.org/wiki/Lamiaceae) | Shrub | Leaves | Chest complaints, cough [15,40] | Alkaloids, essential oils [329] | Methanol: chloroform aerial parts extract showed activity against K. *pneumoniae*, *B. cereus*, *S. aureus* and *M. tuberculosis* with MIC values of 0.5 to 3.0 mg/ml [330] |
| *Salvia officinalis* L. | Lamiaceae | Shrub | Not specified | Sore throat [15] | Alkaloids, essential oils [329] | Ethanol leaf extract showed activity against *B. subtilis*, *S. aureus* and *S. mutans* with MIC value of 3.4 to 4.5 mg/ml [331] |
| *Sansevieria hyacinthoides* (L.) Druce | Asparagaceae | Herb | Leaves | Cough, TB [34] | Flavonoid, steroids [332] | Dichloromethane: methanol leaf extract showed activity against *C. neoformans* with MIC value of 1.7 mg/ml [26] |
| *Scabiosa albanensis* L. | Dipsacaceae | Herb | Leaves, roots | TB [6] | - | - |
| *Scadoxis puniceus* (L.) Friis & Nordal | Amaryllidaceae | Herb | Roots | Blocked nose, chest pain, fever [26] | - | Dichloromethane bulb extract showed activity against *B. subtilis*, *K. pneumoniae* and *S. aureus* with MIC value of 1.6 to 3.13 [333] |
| *^ӿ^Schinus* *molle* L. | Anacardiaceae | Tree | Leaves | Fever [33,168] | Monoterpene hydrocarbons, oxygenated monoterpene hydrocarbon [334] | Dichloromethane bark resin extract showed activity against *S. aureus* and *B. subtilis* with MIC value of 0.13 to 8.0 mg/ml [334] |
| *Samolus valerandi* L. | Primulaceae | Herb | Whole plant | Lung infections [15] | - | - |
| *^α^Sclerocarya* *birrea* (A. Rich.) Hochst. | Anacardiaceae | Tree | Bark | Cold, fever, TB, cough, runny nose [1,26,66,132] | Saponins, tannin [335] | Methanol bark extract showed activity against *S. aureus*, *P. aeroginosa* with MIC value of 0.46 to 0.57 mg/ml [335] |
| [*Schotia brachypetala* Sond.](http://www.ipni.org/ipni/idPlantNameSearch.do?id=518039-1&back_page=%2Fipni%2FeditSimplePlantNameSearch.do%3Ffind_wholeName%3DSchotia%2Bbrachypetala%26output_format%3Dnormal) | Fabaceae | Tree | Bark | TB, cold, fever [17,66] | Flavonolacyl glucosides [336] | Acetone bark extract showed activity against *M. tuberculosis* with MIC value of 25 mg/ml [66] |
| *Scilla natalensis* Planch. | Hyacinthaceae | Herb | Bulb | Sore throat [1,337] | Bufadienolides, saponins [338] | - |
| [*Searsia burchellii* (Sond. ex Engl.) Moffett](http://www.ipni.org/ipni/idPlantNameSearch.do?id=77088406-1&back_page=%2Fipni%2FeditSimplePlantNameSearch.do%3Ffind_wholeName%3DSearsia%2Bburchellii%2B%26output_format%3Dnormal) | Anacardiaceae | Tree | Leaves, whole plant | Cold, cough, fever, influenza [26] | - | Methanol bark extract showed activity against *K. pneumoniae*, *P. aeruginosa* and *S. aureus* with MIC value of 156.25 μg/ml [5] |
| ^α^*Securidaca longepedunculata* Fresen. | Polygalaceae | Tree | Bark, roots | TB, chest complaints [66,339] | Alkaloids, flavonoids, tannins, saponins [335] | Methanol root showed activity against *S. aureus* with MIC value of 0.17 to 0.29 mg/ml [335] |
| *Senecio serratuloides* DC. | Asteraceae | Shrub | Leaves | Chest complaints, blocked and runny nose, cough, fever, sore throat [1,26] | Flavonoid, phenolic compounds, tannin [340] | Dichloromethane: methanol leaf extract showed activity against *C. neoformans* with MIC value of 0.83 mg/ml [26] |
| *Senecio cinerascens* Aiton | Asteraceae | Shrub | Leaves | Cold [22] | - | - |
| *Senecio deltoideus* Less. | Asteraceae | Herb | Leaves | Chest pain, cough, fever, runny nose [26] | - | Dichloromethane: methanol leaf extract showed activities against *C. neoformans*, *K. pneumoniae* and *M. smegmatis* with MIC values of 0.83 to 1.33 mg/ml [26] |
| *Senna italica* Mill. | Fabaceae | Herb | Stem | Chest complaints [67] | Alkaloids, flavonoids, steroids [341] | N-hexane leaf extract showed activity against *S. pneumoniae*, *P. aeruginosa* and *S. aureus* with 20.0 to 29.3 mm zone of inhibition [341] |
| *Silene undulata* L. | Caryophyllaceae | Herb | Leaves | TB [6] | - | - |
| ^#^[*Siphonochilus* *aethiopicus* (Schweinf.) B.L.Burtt](http://www.ipni.org/ipni/idPlantNameSearch.do?id=911455-1&back_page=%2Fipni%2FeditSimplePlantNameSearch.do%3Ffind_wholeName%3DSiphonochilus%2Baethiopicus%26output_format%3Dnormal) | Zingiberaceae | Herb | Tuber | Asthma, cold, cough, influenza [8, 161, 239, 342, 343] | Furanoterpenoids [374] | Petroleum ether root extract showed activity against *K. pneumoniae* and *S. aureus* with MIC values of 0.39 mg/ml [80] |
| *Solanum aculeatissimum* Jacq. | Solanaceae | Shrub | Not specified | Cough, TB [34] | - | - |
| *Solanum giganteum* Jacq. | Solanaceae | Tree | Leaves | Sore throat [1] | - | - |
| *Solanum panduriforme* E. Mey. | Solaneceae | Herb | Leaves | TB [66] | - | Methanol fruit extract showed activity against *S. epidermidis* with MIC value of 2.0 mg/ml [176] |
| *Sonchus asper* (L.) Hill. | Asteraceae | Herb | Leaves | Fever [33] | Alkaloids, flavonoids, phenols, tannins, saponins [344] | Acetone leaf extract showed activity against *S. aureus* and *B. cereus* with MIC value of 2.0 to 5.0 mg/ml [345] |
| *Sonchus dregeanus* DC. | Asteraceae | Herb | Not specified | Cough, TB [34] | - | - |
| *Spermacoce natalensis* Hochst. | Rubiaceae | Herb | Bark | Chest complaints [189] | - | - |
| [*Stachys rugosa* Lam.](http://www.ipni.org/ipni/idPlantNameSearch.do;jsessionid=321278BB9AFA6B14AADFB5016CFE697D?id=459929-1&back_page=%2Fipni%2FeditSimplePlantNameSearch.do%3Bjsessionid%3D321278BB9AFA6B14AADFB5016CFE697D%3Ffind_wholeName%3DStachys%2Brugosa%2B%26output_format%3Dnormal) | Lamiaceae | Shrub | Leaves | Cold, fever, influenza [22] | - | - |
| [*Strychnos decussata* (Pappe) Gilg](http://www.ipni.org/ipni/idPlantNameSearch.do?id=50426037-2&back_page=%2Fipni%2FeditSimplePlantNameSearch.do%3Ffind_wholeName%3DStrychnos%2Bdecussata%2B%26output_format%3Dnormal) | Loganiaceae | Tree | Bark | Fever, sore throat [339] | Alkaloids [346] | - |
| [*Sutherlandia frutescens* (L.) R.Br. ex W.T.Aiton](http://www.ipni.org/ipni/idPlantNameSearch.do?id=157207-3&back_page=%2Fipni%2FeditSimplePlantNameSearch.do%3Ffind_wholeName%3DSutherlandia%2Bfrutescens%2B%26output_format%3Dnormal) | Fabaceae | Shrub | Leaves, stem, whole plant | Cold, fever, influenza, TB [16,81,347] | Flavonol glycosides, L-canavanine, D-pinitol, saponins, triterpenoid glycoside, γ-amino butyric acid [349] | Hexane extract showed activity against *S. aureus* with MIC values of 0.31 mg/ml [348] |
| *Symphytum offinale* L. | Boraginaceae | Shrub | Leaves | Bronchitis, pneumonia [33] | Alkaloids, flavones, flavonoids, saponins, tannins, terpenoides [350] | - |
| *Syzygium cordatum* Hochst. ex C. Krauss. | Myrtaceae | Tree | Bark | Cold, TB, blocked nose, cough, fever, runny nose [26,67,132] | Arjunolic acid, epi-friedelinol, friedelin, glucose, leucodelphinidin, leucocyanidin, β-sitosterol [351] | Dichloromethane: methanol bark extract showed activity against *C. neoformans*, *M. catarrhalis* and *S. aureus* with MIC value of 0.38 to 0.83 mg/ml [26] |
| *Syzygium gerrardii* (Harv. ex Hook. f.) Burtt Davy | Myrtaceae | Tree | Bark | Chest pain, cough, TB [184] | - | - |
| [*Tabernaemontana elegans* Stapf](http://www.ipni.org/ipni/idPlantNameSearch.do?id=82109-1&back_page=%2Fipni%2FeditSimplePlantNameSearch.do%3Ffind_wholeName%3DTabernaemontana%2Belegans%26output_format%3Dnormal) | Apocynaceae | Tree | Roots | TB, chest complaints [12] | Alkaloids, amines, essentials oils, phenols, steroids [352] | Crude extract showed activity against *B. subtilis*, *M. tuberculosis* and *S. aureus* with MIC values of 64 to 128 μg/ml [352] |
| *Tarchonanthus camphoratus* L. | Asteraceae | Tree | Leaves | Asthma, bronchitis [15] | Camphor, flavonoids 1,8-cineole, α-terpineol, pinocembrin [118] | Aerial parts extracts showed activity against *S. aureus,* *B. cereus*, *K. pneumoniae* and *C. neoformans* with MIC values of 0.23 to 0.93 mg/ml [292] |
| [*Tecoma capensis* Lindl.](http://www.ipni.org/ipni/idPlantNameSearch.do?id=103536-3&back_page=%2Fipni%2FeditSimplePlantNameSearch.do%3Ffind_wholeName%3DTecoma%2Bcapensis%26output_format%3Dnormal) | Bignoniaceae | Herb | Roots | Fever, pneumonia [17] | Alkaloids, anthraquinone glycosides, carbohydrates, cardiac glycosides, flavonoids, saponins [353] | - |
| *Tetradenia riparia* (Hochst.) Codd. | Lamiaceae | Shrub | Leaves | Influenza, sore throat, blocked and runny nose, chest pain, chills, cough, fever [16,26] | Alkaloids, flavonoids, phenol, phlobatannins, saponins, steroids [354] | Dichloromethane: methanol leaf extract showed activity against *C. neoformans, M. catarrhalis* and *S. aureus* with MIC value of 0.03 to 0.60 mg/ml [26] |
| *Teucrium africanum* Thunb. | Lamiaceae | Herb | Whole plant | Sore throat [15] | - | Methanol leaf extract showed activity against *B. cereus, K. pneumonia, M. catarrhalis*, P. aeruginosa, *S. aureus* and *S. pyogenes* with MIC value of 1.0 to 4.0 mg/ml [355] |
| *Terminalia sericea* Burch. ex DC. | Combretaceae | Tree | Bark | Blocked and runny nose, cough, TB [26,66] | Flavonoids, saponins, tannin [335] | Methanol leaf extract showed activity against *S. aureus* with MIC value of 0.11 to 0.29 mg/ml [335] |
| *Thesium scirpoides* A.W. Hill. | Santalanaceae | Shrub | Not specified | Cough, TB [34] | - | - |
| [*Tinospora fragosa* (I.Verd.) I.Verd. & Troupin](http://www.ipni.org/ipni/idPlantNameSearch.do?id=581606-1&back_page=%2Fipni%2FeditSimplePlantNameSearch.do%3Ffind_wholeName%3DTinospora%2Bfragosa%26output_format%3Dnormal) | Menispermaceae | Tree | Twigs | Cough [356] | - | - |
| [*Trema orientalis* (L.) Blume](http://www.ipni.org/ipni/idPlantNameSearch.do?id=143448-3&back_page=%2Fipni%2FeditSimplePlantNameSearch.do%3Ffind_wholeName%3DTrema%2Borientalis%2B%26output_format%3Dnormal) | Ulmaceae | Tree | Roots | Cough [239] | Flavanoids, phytosterols, saponins, tannins, triterpenes, xanthones [357] | Aqueouse bark extract showed activity against *B. subtilis* and *K. pneumoniae* with MIC value of 0.625 mg/ml [358] |
| *Trichilia emetica* Vahl subsp. Emetica | Meliaceae | Tree | Leaves | Blocked and runny nose, chest pain, cough, fever [26] | Catechin, epicatechin, flavanol glycoside, sterols, taxifolin [359] | Dichloromethane: methanol leaf extract showed activity against *C. neoformans* and *S. aureus* with MIC value of 0.27 to 0.83 mg/ml [26] |
| *Tulbaghia acutiloba* Harv. | Amaryllidaceae | Herb | Rhizome | TB [6] | - | - |
| *Tulbaghia alliacea* L.f. | Amaryllidaceae | Herb | Whole plant, rhizome | Fever, influenza, TB [15,95] | - | Chloroform bulb extract showed activity against *M. smegmatis* with MIC value of 2.4 mg/ml [360] |
| *Tulbaghia capensis* L. | Amaryllidaceae | Herb | Bulb | Asthma, cold, flu [361] | - | - |
| *Tulbaghia violacea* Harv. | Amaryllidaceae | Herb | Rhizome | Asthma, cold, fever, chest complaints, cough, influenza, sinusitis, TB, ulceration of the lung [1,6, 15,16,161] | Sulfur compounds [118] | Dichloromethane bulb extract showed activity against *K. pneumoniae* and *S. aureus* with MIC value of 0.195 mg/ml [274] |
| *Turbina oblongata* (E.Mey. ex Choisy) A.Meeuse | Convolvulaceae | Herb | Leaves | Sore throat [1,93] | - | - |
| *^ӿ^Urtica* *urens* L. | Urticaceae | Herb | Whole plant | Chest complaints, sore throat, cough [15] | Flavonoids, phenolic, tannin [362] | Ethanol leaf extract showed activity against *S. aureus*, *S. epidermidis*, *P. aeruginosa* and *B. subtilis* with MIC values of 2 μg/ml [362] |
| *Vangueria infausta* Burch. | Rubiaceae | Tree | Roots, leaves | Chest complaints, cough [12] | Flavonoids, genistein, O-glucoside, polyketide [363] | Acetone extract showed activity against *S. pyogenes*, *B. cereus*, *S. aureus* and *K. pneumoniae* with MIC value of 0.04 to 0.62 mg/ml [364] |
| [*Vepris lanceolata* (Lam.) G.Don](http://www.ipni.org/ipni/idPlantNameSearch.do?id=775496-1&back_page=%2Fipni%2FeditSimplePlantNameSearch.do%3Ffind_wholeName%3DVepris%2Blanceolata%2B%26output_format%3Dnormal) | Rutaceae | Tree | Roots | Influenza [239] | Cinnamic acid, furanoquinoline alkaloids [365] | Hexane stem extract showed activity against *S. aureus* and *P. aeruginosa* with MIC values of 32 mg/ml [366] |
| *Viscum capense* L.f. | Santalaceae | Climber | Leaves, stem | Asthma, TB [16] | - | Methanol stem extract showed activity against *S. aureus* with 12.8 mm zone of inhibition [367] |
| ^#α^[*Warburgia salutaris* (G.Bertol.) Chiov.](http://www.ipni.org/ipni/idPlantNameSearch.do?id=146036-1&back_page=%2Fipni%2FeditSimplePlantNameSearch.do%3Ffind_wholeName%3DWarburgia%2Bsalutaris%26output_format%3Dnormal) | Canellaceae | Tree | Bark, leaves | Cold, lung infections, influenza, sore throat, asthma, pneumonia, cough, TB [6,10,67,339] | Alkaloids, flavonoids, tannins, saponins [335] | Methanol bark extract showed activity against *S. aureus* with MIC value of 0.25 to 0.56 mg/ml [335] |
| *Withania somnifera* (L) Dunal | Solanaceae | Shrub | Leaves, roots | Cough, TB [6,54] | Alkaloids, steroids withanolides [368] | Acetone leaF extract showed activity against *B. subtilis*, *S. pyogenes*, *P. aeruginosa* and *K. pneumoniae* with 11.1 to 35.0 mm zone of inhibition [369] |
| [*Ximenia caffra* Sond.](http://www.ipni.org/ipni/idPlantNameSearch.do?id=608572-1&back_page=%2Fipni%2FeditSimplePlantNameSearch.do%3Ffind_wholeName%3DXimenia%2Bcaffra%26output_format%3Dnormal) | Olacaceae | Tree | Leaves, roots | Cough, TB [66, 339] | Flavonoid, flavonol glycosides, polyphenol [370] | Methanol root extract showed activity against *S. epidermidis* and *S. aureus* with MIC value of 1.42 to 5.66 mg/ml [370] |
| *Xysmalobium parviforum* Harv. ex Scott Elliott | Apocynaceae | Herb | Not specified | Cough, TB (Phangula, 2015) | - | - |
| [*Zantedeschia aethiopica* (L.) Spreng.](http://www.ipni.org/ipni/idPlantNameSearch.do?id=100976-3&back_page=%2Fipni%2FeditSimplePlantNameSearch.do%3Ffind_wholeName%3DZantedeschia%2Baethiopica%26output_format%3Dnormal) | Araceae | Herb | Roots | Asthma, bronchial infection [17] | Triterpenes, phenylpropanoids, sterols [371] | Ethanol and water leaf extract showed activity against *S. pyogenes* and *S. aureus* with MIC values of 0.78 mg/ml [80] |
| *Zanthoxylum capense* (Thunb.) Harv. | Rutaceae | Tree | Leaves, roots | Cold, chest pains, cough, sore throat, bronchial infections, fever, TB [1,9,17,18,119,132, 189, 263] | Resins, essential oil, tannins [372] | Dichloromethane root extract showed activity against *M. smegmatis*, *M. tuberculosis* and *M. bovis* with MIC value of 12.5 to 62.5 μg/ml [373] (Luo et al., 2011) |
| [*Ziziphus mucronata* Willd.](http://www.ipni.org/ipni/idPlantNameSearch.do?id=719359-1&back_page=%2Fipni%2FeditSimplePlantNameSearch.do%3Ffind_wholeName%3DZiziphus%2Bmucronata%2B%26output_format%3Dnormal) | Rhamnaceae | Tree | Bark, leaves, roots | Bronchial infection, chest complaint and fever, TB [9,66, 184, 339,340] | Alkaloids, tannins [372] | Acetone bark extract showed activity against *M. tuberculosis* with MIC value of 25 mg/ml [66] |
| **Key:**  **^α^; List of Protected Tree Species under the South African National Forest Act, 1998 (Act No. 84 of 1998), ^ӿ^;** Exotic species to South Africa, ^#^ (species of conservation concern and included on the South African National Red Data List of Plants) | | | | | | |
